# Single-nucleus RNA sequencing reveals heterogenous microenvironments and specific drug response between cervical squamous cell carcinoma and adenocarcinoma

**DOI:** 10.1016/j.ebiom.2023.104846

**Published:** 2023-10-24

**Authors:** Shitong Lin, Yuanhui Sun, Canhui Cao, Zhixian Zhu, Yashi Xu, Binghan Liu, Bai Hu, Ting Peng, Wenhua Zhi, Miaochun Xu, Wencheng Ding, Fang Ren, Ding Ma, Guoliang Li, Peng Wu

**Affiliations:** a Department of Obstetrics and Gynecology, Union Hospital, Tongji Medical College, Huazhong University of Science and Technology, 1277 Jiefang Avenue, Wuhan, Hubei, 430022, PR China; b Cancer Biology Research Center (Key Laboratory of the Ministry of Education), Tongji Hospital, Tongji Medical College, Huazhong University of Science and Technology, Wuhan, Hubei, China; c Department of Gynecologic Oncology, Tongji Hospital, Tongji Medical College, Huazhong University of Science and Technology, Wuhan, Hubei, China; d National Key Laboratory of Crop Genetic Improvement, Huazhong Agricultural University, Wuhan, 430070, China; e Department of Gynecology, First Affiliated Hospital of Zhengzhou University, Zhengzhou, China; f Agricultural Bioinformatics Key Laboratory of Hubei Province, Hubei Engineering Technology Research Center of Agricultural Big Data, College of Informatics, Huazhong Agricultural University, Wuhan, 430070, China

**Keywords:** snRNA-seq, CSCC, CAde, Microenvironments, Drug response

## Abstract

**Background:**

Cervical squamous cell carcinoma (CSCC) and adenocarcinoma (CAde) are two major pathological types of cervical cancer (CC), but their high-resolution heterogeneity of tumor and immune microenvironment remains elusive.

**Methods:**

Here, we performed single-nucleus RNA sequencing (snRNA-seq) from five CSCC and three CAde samples, and systematically outlined their specific transcriptome atlas.

**Findings:**

We found CD8^+^ T cells in CSCC were more cytotoxic but lower exhausted compared to those in CAde, and phagocytic MRC1^+^ macrophages were specifically enriched in CSCC. Interestingly, we discovered that pro-tumoral cancer-associated myofibroblasts (myoCAFs) and cancer-associated vascular-fibroblasts (vCAFs) were more abundant in CSCC, and further verified their pro-metastatic roles *in vitro*. Furthermore, we also identified some specific chemotherapy drugs for CSCC (Dasatinib and Doramapimod) and CAde (Pyrimethamine and Lapatinib) by revealing their heterogeneity in transcriptomic profiles of malignant epithelial cells, and further verified their specific sensitivity in cell lines and constructed CC-derived organoids. Cell–cell communication networks revealed that the pathways of NRG1-ERBB2, and FN1-ITAG3 were specific for CAde and CSCC, respectively, which may partly explain the specificities of identified chemotherapy drugs.

**Interpretation:**

Our study described the immune heterogeneity and specific cellular interactions between CSCC and CAde, which could provide insights for uncovering pathogenesis and designing personalized treatment.

**Fundings:**

10.13039/501100012166National Key R&D Program of China (2021YFC2701201), 10.13039/501100001809National Natural Science Foundation of China (82072895, 82141106, 82103134, 81903114).

**Research in context**

**Evidence before this study**

Cervical cancer (CC) remains one of the most lethal malignancies in developing countries, and cervical squamous cell carcinoma (CSCC) and adenocarcinoma (CAde) are the two mainly pathologic types. These two pathologic types vary greatly regarding the cancer origin, dynamic changes after human papillomavirus (HPV) infection, lymphatic metastasis, age-of-onset, clinical symptom, etc. Nevertheless, there are no obvious differences in the treatment of these two pathologic types according to the National Comprehensive Cancer Network (NCCN) guidelines, although previous studies have proved that patients with CAde favored poorer prognosis compared to those with CSCC, especially for advanced or recurrent diseases. Previous studies of CC by single-cell RNA sequencing revealed the dynamic cellular and molecular changes of CC after HPV infection or radiotherapy. However, limited CAde samples were included in these studies, and no study has focused on exploring the differences between these two pathological subtypes at single-cell solution, leaving the vast heterogeneity between CSCC and CAde largely uncharted.

**Added value of this study**

In the present study, we performed single-nucleus RNA sequencing on eight CC samples (five CSCC and three CAde), and revealed their differences in abundance and functional status of different immunocytes and fibroblasts in the tumor microenvironment. Furthermore, specific cell–cell communication networks of NRG1-ERBB2, and FN1-ITAG3 were identified for CAde and CSCC, respectively. Sensitive chemotherapy drugs targeting these communication networks were also identified and validated for CSCC (Dasatinib and Doramapimod) and CAde (Pyrimethamine and Lapatinib), respectively.

**Implications of all the available evidence**

This study revealed the heterogeneity of microenvironments and specific cellular interactions between CSCC and CAde, which deepened the understanding of the pathogenesis of CC with different pathological types. Our study could provide insights for exploring specific therapeutic strategies for CSCC and CAde.

## Introduction

Cervical cancer (CC) remains one of the most common gynecological malignancies in developing countries, which results in 604,127 new cases and 341,831 fatalities worldwide in 2020.^1^ Cervical squamous cell carcinoma (CSCC) and adenocarcinoma (CAde) are the two primary pathologic types, accounting for 75–90% and 10–25% of CC cases, respectively.^2^ These two pathologic types vary greatly regarding the cancer origin, dynamic changes after human papillomavirus (HPV) infection, lymphatic metastasis, age-of-onset, clinical symptom, etc. Nevertheless, there are no obvious differences in the treatment of these two pathologic types according to the National Comprehensive Cancer Network (NCCN) guidelines, although previous studies have proved that patients with CAde favored poorer prognosis compared to those with CSCC, especially for advanced or recurrent diseases.^3^ Pembrolizumab has been approved for advanced or recurrent CC, while the differences in immunotherapy efficacy between CSCC and CAde remain unknown for limited CAde samples were included in the KEYNOTE-158 and KEYNOTE-826 trials.^4^^,^^5^ With the popularity of CC screening and prophylactic HPV vaccine, the ratio of CAde/CSCC is gradually increasing. Therefore, it is of great importance to explore specific therapeutic strategies for these different pathologic subtypes by uncovering their great heterogeneity in transcriptomic profiles and tumor microenvironments.

Previous studies have classified CC from The Cancer Genome Atlas (TCGA) cohort into keratin-low squamous, keratin-high squamous, and adenocarcinoma-rich subgroups based on molecular characteristics, and identified specific somatic mutation sites for CSCC (e.g., MAPK1, HLA-B, EP300, FBXW7, NFE2L2, TP53, and ERBB2) and CAde (e.g., ELF3 and CBFB) cohorts.^6^^,^^7^ However, these findings are based on the bulk-RNA/DNA sequencing, which reflects the average changes of whole tumor tissues containing a mixed population of cells. Therefore, the specific genomic and transcriptomic profiles of specific cellular types from these two pathologic types remain elusive. Recently, the emergency of single-cell RNA sequencing is epoch-making, which provides a high-resolution technology for mapping cellular subtypes to uncover the inter-tumoral and intra-tumoral heterogeneity.^8^^,^^9^ With this technology, the dynamic cellular and molecular changes of CC after HPV infection or radiotherapy have been well described.^10^, ^11^, ^12^, ^13^, ^14^, ^15^, ^16^, ^17^, ^18^ Our group has also previously identified a specific cancer-associated fibroblasts (CAFs) subtype surrounding the cancerous foci which play pivotal roles in remodeling suppressive microenvironment in CSCC using single-nucleus RNA sequencing (snRNA-seq) and spatial transcriptomics.^19^ However, limited CAde samples were included in previous studies, and no study has focused on exploring the differences between these two pathological subtypes at single-cell solution, leaving the vast heterogeneity between CSCC and CAde largely uncharted.

To address this unknown issue, we performed snRNA-seq on eight CC samples (five CSCC and three CAde), and 82,719 single-cell transcriptomes were further well characterized. We comprehensively revealed the transcriptomic profiles of malignant epithelial cells, T cells, myeloid cells, and fibroblasts, and compared their heterogeneity regarding the percentage of cellular subtypes, functional status, and cell–cell communications between CSCC and CAde by bioinformatics and experimental methods. Collectively, our study may provide insights for exploring specific pathogenesis and therapeutic strategies for CSCC and CAde by dissecting their heterogeneity at single-cell resolution.

## Methods

### Samples collection

This study was approved by the Medical Ethics Committee of Tongji Medical College, Huazhong University of Science and Technology (TJ-IRB20210609). Samples used for snRNA-seq were collected from eight patients (five with CSCC and three with CAde) diagnosed with CC in the Department of Obstetrics and Gynecology of Tongji Hospital in Wuhan, China. All patients did not receive any other treatments before surgical procedures, and their clinical characteristics were provided in Supplementary Table S1. A tissue microarray containing 32 CSCC and 11 CAde paraffin-embedded tissues was used for further validation, and their detailed clinical characteristics were provided in the Supplementary Table S2.

### snRNA-sequencing

The methods have been described in detail in our previous manuscript.^19^ Briefly, the fresh CC tissues were quick-frozen in liquid nitrogen for 1 h upon receipt of samples and stored in a refrigerator at −80 °C. We used the Chromium Next GEM Single-Cell Multiome ATAC + Gene Expression User Guide (CG000338) to perform Nuclei isolation and permeabilization according to the manufacturers’ recommendations. The Chromium Single Cell 3ʹ Reagent Kits v3 (10 × Genomics, USA) was used to construct the snRNA-seq libraries. High-quality snRNA-seq data was produced after a range of experimental procedures, including cell counting and quality control, gel beads-in-emulsion (GEMs) generation and barcoding, post-GEM-RT cleanup, cDNA amplification, gene expression library construction, and NovaSeq platform (Illumina, USA) sequencing.

### Identification of viral RNA

Viral reads were mapped against 83 HPV reference genomes downloaded from PaVE by BWA (v0.7.13).^20^^,^^21^ The genome coverage (covered length/full length of the reference genome) and effective depth (total mapped bases/covered length) of each type were calculated to determine the infective HPV type of each sample. Only samples with a viral genome coverage >5% and an effective depth >50 × were deemed HPV-positive. The characteristics of HPV alignment were displayed in Supplementary Table S3.

### snRNA-sequencing data processing

Firstly, employing GRCh38 and HPV16 reference genomes, we constructed a custom reference transcriptome using mkref command of Cell Ranger (v6.1.2). Raw snRNA-seq data were demultiplexed, aligned to the custom reference transcriptome, and quantified gene expression matrices using Cell Ranger with default parameters. Next, The R package scCancer (v2.2.1) was applied to filter out cells with low quality for each sample.^22^ The filtering thresholds of all eight samples were summarized in Supplementary Table S4. To remove potential doublets in each sample, we predicted the doublet score of each cell using the R package scCancer and determined the threshold for doublets by catching outliers (Q3 + 1.5 ∗ IQR) from the distribution of doublet scores. After quality control, the R package Seurat (v4.1.1) was used to integrate data from all eight samples by IntegratedData function, which can remove the potential batch effect.^23^ Further analysis, including data normalization, highly variable gene identification, data scaling, and dimension reduction were sequentially performed by Seurat with default parameters.

### Cell-type clustering and major cell-type identification

FindClusters function in Seurat was used with the first 30 principal components and a resolution 0.8 to generate 23 cell clusters composed of 82,719 cells. These cells were then annotated to nine major cell types with following the canonical marker genes: Epithelial cell (CDKN2A, CDH1, EPCAM, MUC5B, WFDC2, PTPRT), Mesenchymal cell (COL1A1, LAMA2, ACTA2), T cell (CD247, CD2, CD3E), Myeloid cell (MSR1, HLA-DPB1), Vascular Endothelial cell (EGFL7, EMCN), Plasma cell (IGKC, MZB1), Lymphatic Endothelial cell (CCL21, PROX1), B cell (MS4A1) and Mast cell (KIT, CPA3).

The clusters assigned to the same cell type were lumped together for the sub-cell type analysis. The selected cells were processed with normalization, highly variable gene identification, data scaling and dimension reduction. Harmony (v0.1.0) was performed to remove potential batch effect except for malignant or epithelial cells.^24^ In the same way, sub-cell clusters were identified by the FindClusters function in Seurat. Cell cycle stage was calculated by CellCycleScoring function in Seurat R package.

### Differentially expressed genes (DEGs) identification and gene functional enrichment

The DEGs of each cluster were generated by FindMarkers or FindAllMarkers function with default parameter and Wilcoxon rank-sum test unless explicitly emphasized. DEGs with adjusted *P*-value less than 0.05 were retained for further analysis. The significant DEGs were identified by following rules: 1) The absolute value of log2 fold change was more than 0.25; 2) Genes were expressed in more than 10% cells of the cluster; 3) The adjusted *P* value was less than 0.05. Enrichment analysis of DEGs was performed by R package ClusterProfiler (v4.2.2) and Metascape (https://metascape.org).^25^^,^^26^ In addition, gene set enrichment analysis (GSEA) was performed by fgsea (v1.20.0). Gene set variation analysis (GSVA) was performed by GSVA package (v1.42.0).^27^ Functional score was calculated by AddModuleScore/AddModuleScore_Ucell function. The gene sets were collected from MSigDB database by msigdbr package. Some known functional gene sets of T cells and macrophages were displayed in Supplementary Table S5.^28^^,^^29^

### Copy number variation (CNV) inferring and malignant cells identification

Non-malignant cells (1000 vascular endothelial cells) employed as reference cells and 8321 candidate malignant cells sampled from epithelial cells (∼20% of epithelial cells) were used to infer the CNVs of eight patients based on the guides of inferCNV (v1.14.0).^30^ The CNV score of each cell was defined as the quadratic mean of expression value in CNV regions. Thus, cells with high CNV scores were identified as malignant cells. The distribution of CNV scores in each cluster of epithelial cells was compared to that of reference cells to identify the malignant cell clusters.

### Trajectory analysis

Single-cell trajectory analysis was conducted using monocle 2 (v2.22.0).^31^ The count matrix from a specific cell type was utilized and genes expressed in less than 10 cells were retained for further analysis. For T or Myeloid cells, ordering genes were determined by dispersion and expression levels of all genes. For fibroblasts, ordering genes were determined by differentialGeneTest function with a q-value less than 1E-10. Finally, the trajectory was constructed by the reduceDimension function with DDRTree method and filtered ordering genes.

After calculating the function scores for T cells (cytotoxicity, exhaustion and Treg score) and DC cells (activated and migratory score), locally weighted scatterplot smoothing (LEOSS) regression was applied to fit function score with trajectory component.

### Cell–cell communication analysis

To understand the communication network among nine major cell types, cell–cell communication analysis was conducted by CellphoneDB (v3.1.0), and only ligand-receptor pairs with *P*-value less than 0.05 were retained.^32^

For Cellchat (v1.4.0) analysis, all the legend-receptor pairs (Secreted Signaling, ECM-Receptor, and Cell–Cell Contact) were used to construct cell–cell communication network between Cancer cells and fibroblasts in two separate pathologic types with default parameters.^33^ The mergeCellChat function was then used to create a merged object and further analysis was conducted according to the guidelines described for multiple datasets.

### Transcription factor activity analysis

The software pySCENIC (v0.10.0) was utilized for gene regulatory network construction.^34^ Activities of the transcription factors were evaluated by AUCell. The calcRss function was used to find the specific transcription factor in CAde and CSCC, respectively. The gene regulatory network was visualized by Gephi software (v0.9.2).

### TCGA survival analysis

Kaplan–Meier overall survival analysis was performed using the TCGA CC cohort. The mean expression value of the gene signatures was calculated. To determine the optimal low and high cutoff points for the expression level of gene signatures, we utilized the “surv_cutpoint” function from the survival R package (v3.4-0). Subsequently, a Cox proportional hazards regression model was constructed using the “coxph” function from the survival R package. Comprehensive characteristics of CC samples from the TCGA cohort were presented in Supplementary Table S6.

### Drug response in cancer cells

To analyze the different responses to drugs in cancer cells between CAde and CSCC, Beyondcell (v1.3.3) was performed to CAde and CSCC cancer cells separately.^35^ Drug Sensitivity Signatures collection (SSc) that captured the sensitivity to the given drug was selected for analysis. The beyondcell score for each cell-drug pair and the switch point (SP) for each drug were calculated by bcScore function with default parameters. In brief, SP represents the points at which cells switch from a down-regulated status to an up-regulated status (in other words, from sensitivity to resistance). Thus, the homogeneous drug response for cancer cells with the same directionality to a certain drug response would be represented by an extremum, either toward sensitive (SP = 0) or resistance (SP = 1), while a heterogeneous drug response for cancer cells would be represented by an intermediate SP.

### Calculation of cancer stemness

In our study, the cancer stemness score was calculated by runStemness function in scCancer package.^22^^,^^36^ In brief, Spearman correlation between the scale data of single-cell expression profile and stemness signature vector was calculated, and the min–max normalized coefficient was defined as the final stemness score.

### Calculation of pearson correlation coefficient between different fibroblast clusters

Pearson correlation coefficients was calculated based on normalized enrichment score (NES) matrix (row: pathway, col: cell clusters, value: NES) calculated by GSEA analysis. In brief, we used the presto R package (v1.0.0) (https://github.com/immunogenomics/presto) to rank the different expression gene of each cluster by “Log2FC value”. Then GSEA analysis were employed to calculated the NES of each cluster on REACTOME pathway. Finally, we calculated the Pearson correlation coefficients based on NES matrix.

### Immunohistochemistry (IHC) and multi-color immunofluorescence (mIF)

The operational methods of IHC and mIF have been described in detail in our previous research.^19^ Briefly, a tissue microarray containing 32 CCSC and 11 CAde samples was used to validate our findings from scRNA-seq. The details of primary antibodies used in this study were as follows: MTSS1 (Atlas Antibodies, #HPA075540, 1:200), POLA1 (Atlas Antibodies, #HPA002947, 1:200), POSTN (Abcam, #ab215199, 1:500), COL1A1 (CST, #72026, 1:100), LMCD1 (Immunoway, #YT7409, 1:100), MRC1 (CST, #91992, 1:100), CD68 (MXB, #kit-0026, 1:1), PROX1 (Proteintech, #67834-1-Ig, 1:250), SOD2 (Proteintech, #CL594-66474, 1:100) (Supplementary Table S7). The method for measuring the estimated staining score of targeted proteins was described in our previous study according to the positivity percentage and staining intensity.^19^

### Culture of CC cell lines and human umbilical vein endothelial cell (HUVEC)

CC cell lines SiHa (RRID: CVCL_0032), HeLa (RRID: CVCL_0030), CaSki (RRID: CVCL_1100), and ME-180 (RRID: CVCL_1401) were purchased from the American Type Culture Collection (ATCC), and cultured with complete DMEM (Gibco, #11965118) containing 10% FBS (EVERY GREEN, #11011-8611) and 1% penicillin-streptomycin (Gibco, #15140163). Primary HUVEC was purchased from the company of iCell Bioscience Inc (#h110) and cultured with ECM (ScienCell, #1001) containing 10% FBS (EVERY GREEN, #11011-8611) and 1% penicillin-streptomycin (Gibco, #15140163). All cells were cultured in an incubator at 5% CO2 and 37 °C.

### Extraction of primary fibroblasts and flow cytometry sorting

The tumor tissue of CC samples was separated into tissue blocks of 1 mm in size under sterile conditions, and then dispersed and digested with collagenase, hyaluronidase and DNase I at 37 °C for 3 h. The supernatant was discarded after centrifugation at 1000 rpm for 5 min, and the supernatant was resuspended with red blood cell lysate and centrifuged again after 3 min. The primary fibroblasts were resuspended with sterile PBS and cultured with DMEM/F12 medium (Gibico, #12634010) containing 10% FBS (EVERY GREEN, #11011-8611) and 1% penicillin-streptomycin (Gibco, #15140163) in an incubator at 5% CO2 and 37 °C. Primary cells underwent at least three rounds of digestion and passage to obtain high-purity primary fibroblasts. Then primary fibroblasts were incubated in staining cocktail containing anti-COL1A1 (Proteintech, #14695-1-AP), anti-POSTN (Proteintech, #66491-1-Ig) and anti-SOD2 (Proteintech, #CL594-66474) in FACS buffer for 30 min on ice (Supplementary Table S7). We gated on POSTN^+^/COL1A1^+^ and SOD2^+^/COL1A1^+^ cells after excluding doublets, respectively.

### Preparation of conditioned medium (CM)

To obtain CM, primary myoCAFs and vCAFs were seeded into 10 cm Petri dish for 48 h. The collected supernatant was centrifuged at 1000*g* for 10 min, and filtered with 0.45 μm filters.

### Measurement of half maximal inhibitory concentration (IC50)

1500 CC cells were seeded into 96-well plates for 24 h. Then different concentrations of identified drugs were added for 24 h, and OD450 values were measured using the Cell Counting Kit-8 (CCK-8, Dojindo Laboratories, #CK04). The details of drugs were as follows: Pyrimethamine (MCE, #HY-18062), Lapatinib (MCE, #HY-50898), Dasatinib (MCE, #HY-10181), and Doramapimod (MCE, #HY-10320). The IC50 values of different drugs were calculated with the GraphPad Prism 6.

Organoids were dissociated into single cells using TrypLE two days prior to the onset of drug exposure and filtered using a 70-mm nylon cell strainer. 2000 cells were sown into 96-wells plate and cultured for 5 days to develop into organoids. Then, Lapatinib (MCE, #HY-17394), Dasatinib (MCE, #HY-17393), Pyrimethamine (MCE, #HY-17026) and Doramapimod (MCE, #HY-10320) were added at various concentrations. ATP levels were measured using the CellTiter-Glo 3D Viability Assay (Promega, #G9683) in accordance with the manufacturer's instructions, and luminescence was assessed using a Synergy 2(BioTek) two days (48 h) following the addition of the medications.

### Migration assay

40,000 CC cells (SiHa, HeLa, CaSki, and ME-180) suspended with 200 μl serum-free DMEM were added into the upper chamber of transwell chamber (Corning, #3422), and 500 μl CM was added into each lower chamber. The upper chambers were fixed with 4% paraformaldehyde for 10 min and stained with 0.5% crystal violet for 30 min.

### Blood vessel formation assay

100 μl Matrigel (Corning, #356243) was added into each 96-wells plate, and the plate was placed in the incubator for 30 min for gel coagulation. 20,000 HUVECs suspended with 100 μl CM from different primary fibroblasts or fresh DMEM containing 10% FBS (Control group) were added into each 96-wells plate. Related images were then obtained every 2 h using an inverted microscope.

### Tumor-derived organoid (tumoroid) culture

The detailed characteristics of collected CC samples for constructing organoids were displayed in Supplementary Table S2. The cervical tumor tissues were first mechanically minced by a scalpel and then digested in a collagenase solution (2 mg/ml of collagenase I, Solarbio, #C8140) at 37 °C for 40–60 min. After the digestion process, cells were filtered through the 70 μm nylon cell strainer and centrifuged at 400*g* for 5 min. The cells were then thoroughly mixed with Basement Membrane Extract (Cultrex Reduced Growth Factor BME, Type 2, R&D, #3533-010-02), 35 μl drop containing 5000–10,000 cells drop was seeded vertically onto the 24-wells plates. The culture plate was solid in a 37 °C cell incubator for 30 min, and then the appropriate medium was added. The medium for CSCC/CAde-organoid consisted of advanced DMEM/f12 (Gibico, #12634010) supplemented with 1 × Hepes (Boster, #PYG0019), 1 × Penicillin/Streptomycin/Amphotericin B, sterile solution (Solarbio, #P7630), 1 × Glutamax (Thermo Fisher Scientific, #35050061), 1x Mycoplasma Elimination Reagent (Yeasen, #40607ES03), 100 ng/ml Recombinant Human Noggin (PeproTech, #120-10C), 200 ng/ml Fibroblast growth factor 7 (PeproTech, #100-19), 100 ng/ml Fibroblast growth factor 10 (PeproTech, #100-26), 1 μM SB202190 (MCE, #HY-10295), 2.5 mM Nicotinamide (MCE, #HY-B0150), 1.25 mM N-acetylcysteine (MCE, #HY-B0215), 10 μM Forskolin (MCE, #HY-15371), 1 × B-27 (Gibico, #17504044), 10 μM Y-27632 (MCE, #HY-10583), 500 nM A83-01 (Selleck, #S7692).

### RNA extraction, PCR and qRT-PCR

The total RNA of primary fibroblasts was extracted using the Trizol reagent (Invitrogen, #15596026), and the cDNA was synthesized using the HiScript II Q RT Super Mix (Vazyme, #R223-01). GAPDH was used as the reference. The primer sequences of target genes were displayed in Supplementary Table S8.

### Statistical analysis

R v4.0.3 was used for statistical analysis. Student's t-test and Wilcoxon rank-sum test were conducted to determine the statistical significance. Calculated *P* < 0.05 was considered statistically significant.

### Ethics approval

This study was reviewed and approved by the Medical Ethics Committee of Tongji Medical College, Huazhong University of Science and Technology (TJ-IRB20210609). Written informed consents had been obtained from all participants.

### Role of funders

All the funders played no roles in study design, data collection, data analysis or writing of report.

## Results

### Single-cell transcriptome landscape of CSCC and CAde

**Fig. 1:**
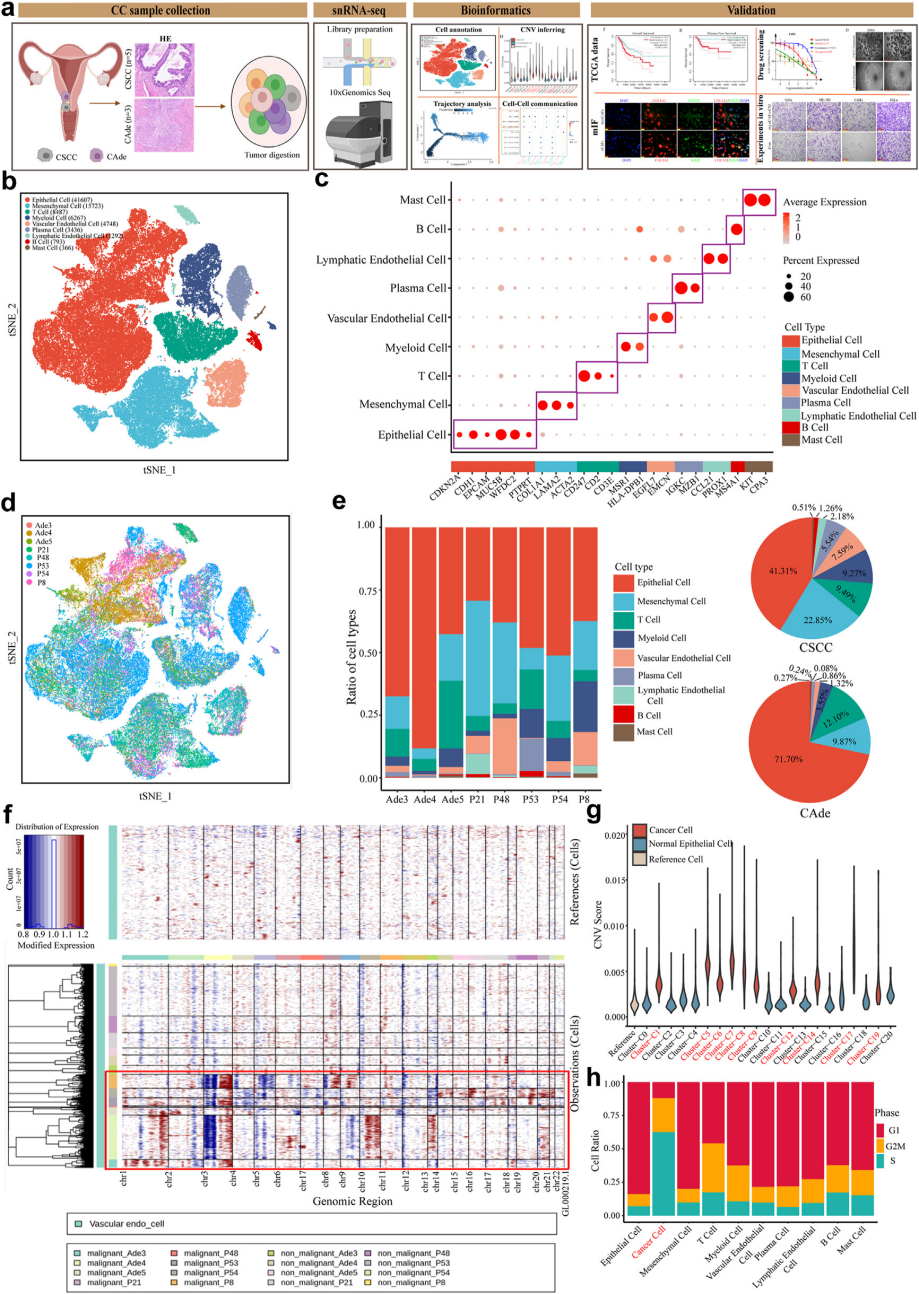
**Construction of single-cell expression atlas for cervical squamous cell carcinoma (CSCC) and adenocarcinoma (CAde) tissues. (a)** The study design and flowchart. **(b)** t-distributed stochastic neighbor embedding (tSNE) plots displayed the nine major cell types of cervical tissues based on snRNA-seq. **(c)** Dot plots displayed the normalized expression levels of canonical markers in identified cell types. **(d)** t-SNE plots of all identified single cells, colored by individual samples. **(e)** The proportions of cell types in each individual sample (left) and pathologic types (right). **(f)** Inferring of copy number variation (CNV) of epithelial cells. The upper panel exhibited large-scale CNVs of single cells inferred based on vascular endothelial cells (1000 cells). The lower panel exhibited large-scale CNVs of single cells inferred based on epithelial cells (∼20% epithelial cells). **(g)** Violin plots displayed the CNV score of each epithelial cell subcluster. **(h)** The cell cycle status of each identified cell type.

**Supplementary Figure S1:**
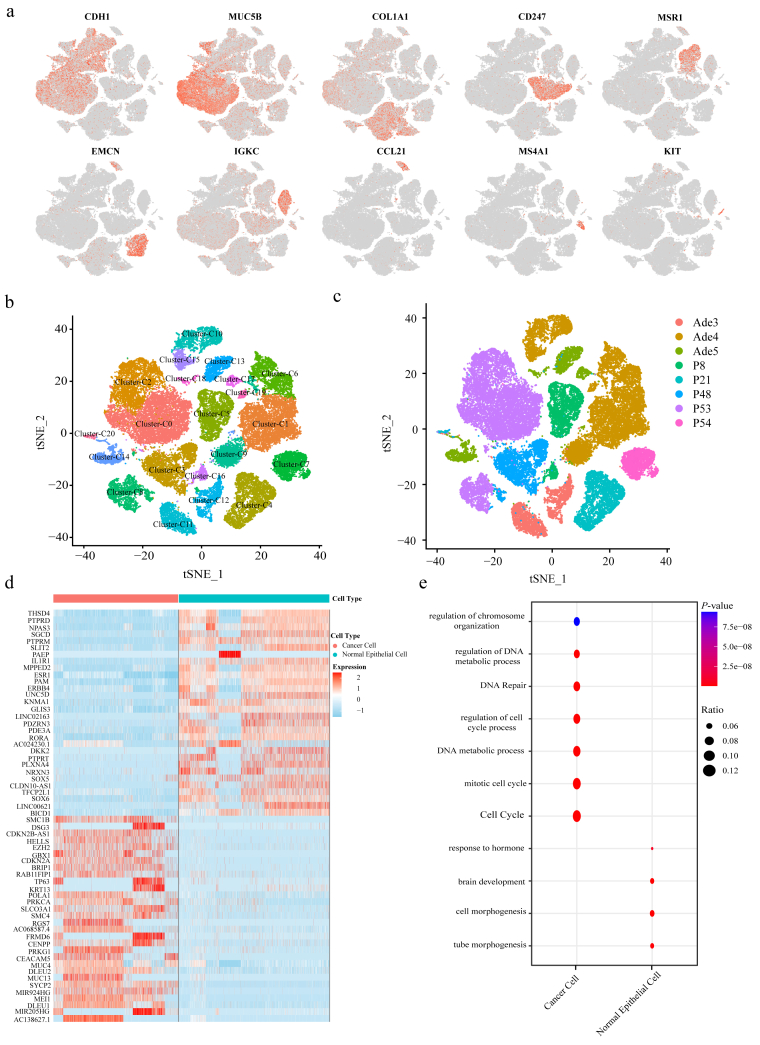
Characteristics and subclusters of epithelial cells in CSCC and CAde. (a) t-SNE plots of all identified single-cells, colored by marker gene expression. **(b–c)** t-SNE plots of all epithelial cells, colored by subclusters and individual samples, respectively. **(d)** Heatmap displayed the expression profiles of top 30 differentially expressed genes in normal epithelial cell and cancer cell. **(e)** Dot plots displayed the GO enrichment terms of normal epithelial cell and cancer cell.

To decipher the inter-tumoral and intra-tumoral heterogeneity between CSCC and CAde, we conducted snRNA-seq on five CSCC samples (P8, P21, P48, P53, P54) and three CAde samples (Ade3, Ade4, and Ade5) (Fig. 1a). The clinical characteristics of included patients were displayed in Supplementary Table S1. After data quality control and filtering, we collected a total of 82,719 single cells, with an average of 1854 genes captured per cell. Based on the canonic marker gene, all these single cells were annotated as nine major cell populations: epithelial cells (CDKN2A, CDH1, EPCAM, MUC5B, WFDC2, and PTPRT), mesenchymal cells (COL1A1, LAMA2, and ACTA2), T cells (CD247, CD2, CD3E), myeloid cells (MSR1 and HLA-DPB1), vascular endothelial cells (EGFL7 and EMCN), plasma cells (IGKC and MZB1), lymphatic endothelial cells (CCL21 and PROX1), B cells (MS4A1), and Mast cells (KIT and CPA3) (Fig. 1b and c and Supplementary Figure S1a). The identified DEGs of each cell cluster were displayed in Supplementary Table S9 (|Log2FC| > 0.25, *P* < 0.05, Wilcoxon Rank Sum Test). Epithelial cell was the most abundant cell type both in CSCC and CAde samples. The fractions of nine cell types in different individuals varied greatly, which indicated the huge inter-individual heterogeneity in CC (Fig. 1d and e).

To further explore the heterogeneity of epithelial cells between CSCC and CAde, we first divided the epithelial cells into 21 distinct clusters (Supplementary Figure S1b), and their respective sample origins were displayed in Supplementary Figure S1c. We measured the relative CNV score of each epithelial cluster compared to reference cell (vascular endothelial cells), and cluster-1/5/6/7/8/9/12/14/17/19 had higher CNV scores compared to reference cells, which were classified as malignant epithelial cells (Fig. 1f and g). We also measured the cell cycle status of each cell type, and observed that the cancer cells showed the lowest percentage of G1 phase, which indicated most cancer cells were more active in DNA replication (Fig. 1h). The top 30 DEGs (|Log2FC| >0.25, *P* < 0.05, Wilcoxon Rank Sum Test) of normal epithelial cell and cancer cell were displayed in Supplementary Figure S1d, and functional enrichment analysis revealed that the pathways of cell cycle, mitotic cell cycle, DNA repair, and DNA metabolic process were active in cancer cell. While normal epithelial cell mainly got involved in the processes of response to hormone, brain development, cell morphogenesis, and tube morphogenesis (Supplementary Figure S1e).

### Different proportions and functional status of T cells and macrophage subcluster in CSCC and CAde

**Fig. 2:**
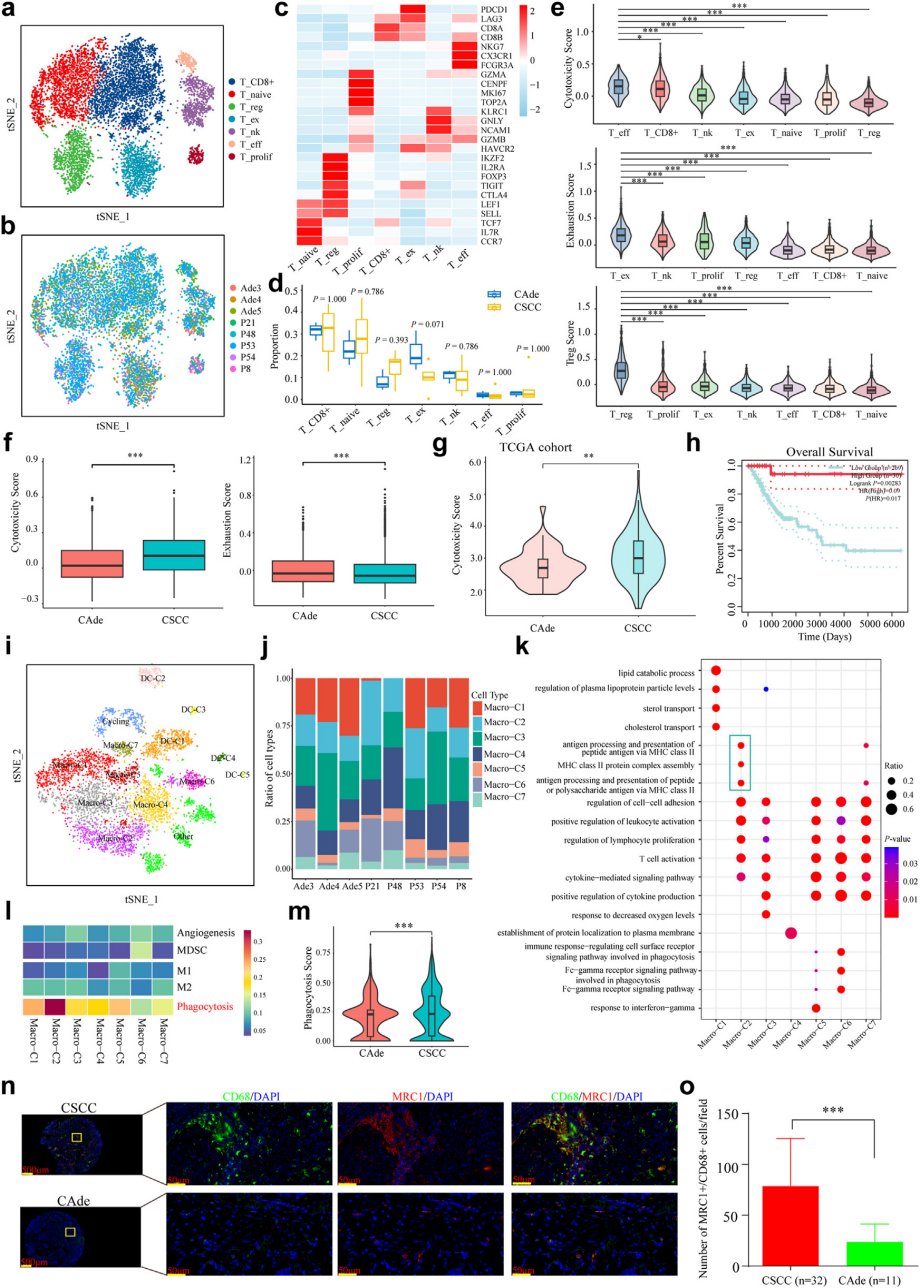
**Characteristics and functional status of T cells and macrophages in CSCC and CAde. (a**–**b)** t-SNE plots of the T cells, colored by cell type and individual samples, respectively. **(c)** Heatmap displayed the expression profiles of marker genes in each identified T cell subcluster. **(d)** Box plots displayed the composition comparison of each T cell subcluster in CSCC and CAde. The *P*-values were calculated by Wilcoxon Rank Sum Test. **(e)** Violin plots depicted the estimated scores of different functional statues in identified T cell subclusters. ∗*P* < 0.05, ∗∗∗*P <* 0.001. The *P*-values were calculated by Wilcoxon Rank Sum Test. **(f)** Box plots displayed the cytotoxicity (left) and the exhausted (right) score of CD8^+^ T cells in CSCC and CAde. ∗∗∗*P* < 0.001. The *P*-values were calculated by Wilcoxon Rank Sum Test. **(g)** Violin plot indicated the cytotoxicity scores of CSCC and CAde from the TCGA cohort. ∗*P* < 0.05. The *P*-value was calculated by Wilcoxon Rank Sum Test. **(h)** Kaplan–Meier curve of overall survival based on the gene signature of cytotoxicity in all CC cohort from the TCGA database. The *P*-value was calculated by Log-rank test. **(i)** t-SNE plots of the myeloid cells, colored by cell type. **(j)** The fraction of each macrophage subcluster in eight individual samples. **(k)** Dot plots depicted the enrichment GO terms of each identified macrophage subcluster. **(l)** Heatmap displayed the functional activity scores of each macrophage subcluster based on known gene signatures. **(m)** Violin plot depicted the phagocytosis scores of CSCC and CAde. ∗∗∗*P* < 0.001. The *P*-value was calculated by Wilcoxon Rank Sum Test. **(n)** mIF staining of macrophages with phagocytotic function in collected CSCC (n = 32) and CAde (n = 11) samples. **(o)** Box plots displayed the number of Macro-C2 (MRC1^+^/CD68^+^) cells/field in CSCC and CAde. ∗∗∗*P* < 0.001. The *P*-value was calculated by Wilcoxon Rank Sum Test.

**Supplementary Figure S2:**
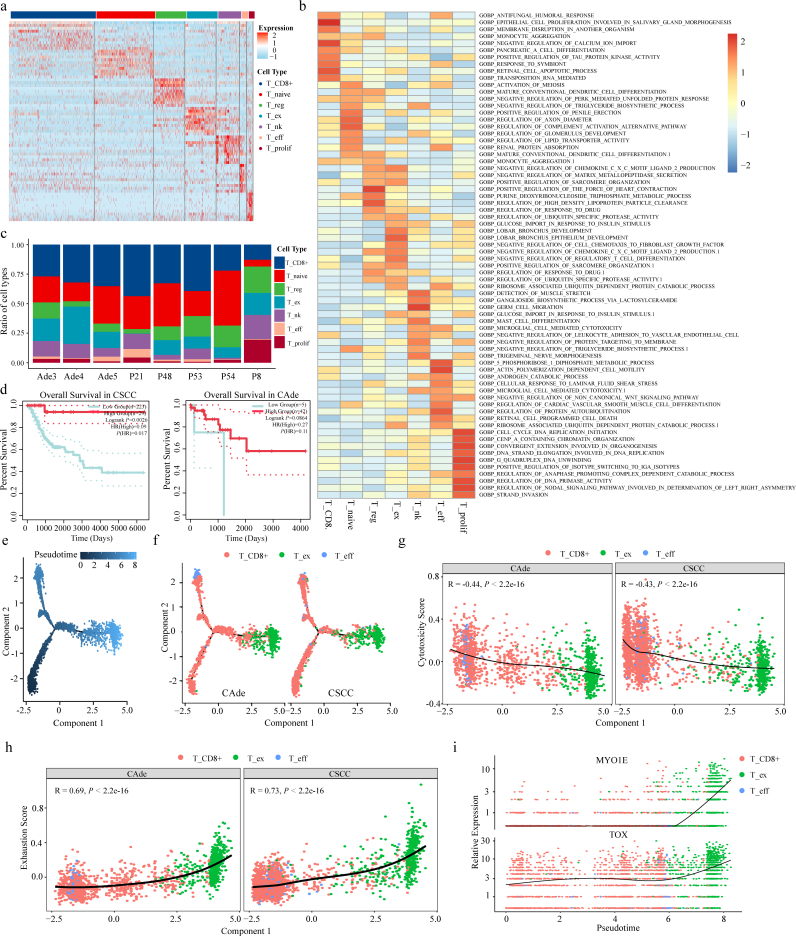
Characteristics of T cells in CSCC and CAde. (a) Heatmap presented the expression profiles of markers genes in each identified T cell subcluster. **(b)** Heatmap displayed the identified GO enrichment terms of each identified T cell subcluster based on GSVA analysis. Color reflected the z-score. **(c)** Bar plot displayed the proportion of each subcluster in individual samples. (**d**) Kaplan–Meier curves regarding the overall survival based on the gene signature of cytotoxicity in CSCC cohort (left) and CAde cohort (right) from the TCGA cohort. The *P*-values were calculated by Log-rank test. **(e–f)** Developmental trajectory of CD8^+^ T cells constructed by monocle 2, colored by pseudo-time and pathologic types, respectively. **(g–h)** The cytotoxic and exhausted scores of CAde and CSCC changed dynamically with pseudo-time, respectively. The solid lines represented LOESS fitting of the relationship between estimated scores and Monocle component 1. *P*-value was calculated by t-test. **(i)** Dynamic changes of representative marker genes during the pseudo-time in CD8^+^ T cells.

T cells are the major component of the tumor microenvironment which plays pivotal roles in antitumor, and we aimed to further dissect the heterogeneity of T cells regarding subpopulations and functional status between CSCC and CAde.^37^^,^^38^ T cells were further divided into seven clusters based on their marker genes, namely T_CD8^+^ (CD8A and CD8B), T_naive (TCF7, IL7R, and CCR7), T_reg (IKZF2, IL2RA, and FOXP3), T_ex (PDCD1, and LAG3), T_nk (GNLY and NCAM1), T_eff (NKG7,CX3CR1 and FCGR3A), T_prolif (CENPF, MKI67, and TPO2A) (Fig. 2a–c and Supplementary Figure S2a). The identified DEGs of each T cells cluster were displayed in Supplementary Table S10 (|Log2FC| > 0.25, *P* < 0.05, Wilcoxon Rank Sum Test). The corresponding functions of each cluster measured by GSVA analysis were displayed in Supplementary Figure S2b. The distributions of each cluster in individual sample also varied greatly, which indicated the huge heterogeneity and complexity of the microenvironment in CC (Supplementary Figure S2c). Overall, T_CD8^+^ and T_naive were abundant in CSCC and CAde. While the propotion of T_reg seemed to be higher in CSCC, and the proportion of T_ex was higher in CAde, although *P*-values (Wilcoxon Rank Sum Test) was not statistically significant (Fig. 2d). We also measured the cytotoxicity/exhaustion/treg score of each cluster to evaluate the functional status of T cells, with T_eff, T_ex, and T_reg obtaining the highest cytotoxic, exhausted, and treg scores, respectively (Fig. 2e). We then further compared the differences in cytotoxic and exhausted function between these two pathologic types. As shown in Fig. 2f, CSCC obtained a higher cytotoxic score but a lower exhausted score compared to CAde. Patients with CSCC from TCGA cohort were also confirmed to have higher cytotoxic score compared to patients with CAde (Fig. 2g), and patients with higher cytotoxic score favored better prognosis in all CC cohort (Log-rank *P* = 0.00283, Log-rank test), CSCC cohort (Log-rank *P* = 0.0026, Log-rank test) from the TCGA database, but not in the CAde cohort (Log-rank *P* = 0.0864, Log-rank test) (Fig. 2h and Supplementary Figure S2d).

We wondered whether the functional changes of T cells in CSCC and CAde underwent the same developmental process, further development trajectory inference reconstructed by the Monocle2 algorithm displayed similar developmental trajectories between these two pathological types, which dynamically derived from the T_CD8^+^ cells and gradually differentiated into T_eff and T_ex (Supplementary Figure S2e and f). The cytotoxic score gradually decreased in CAde (R = −0.44, *P* < 2.2e-16, t-test) and CSCC (R = −0.43, *P* < 2.2e-16, t-test), while the exhausted score increased along with the developmental trajectory in CAde (R = 0.69, *P* < 2.2e-16, t-test) and CSCC (R = 0.73, *P* < 2.2e-16, t-test) (Supplementary Figure S2g and h). The key transcription factor (TOX), and the marker gene (MYO1E) of exhausted CD8^+^ T cells were gradually up-regulated with the pseudo-time, which was consistent with previous studies (Supplementary Figure S2i).^39^, ^40^, ^41^

**Supplementary Figure S3:**
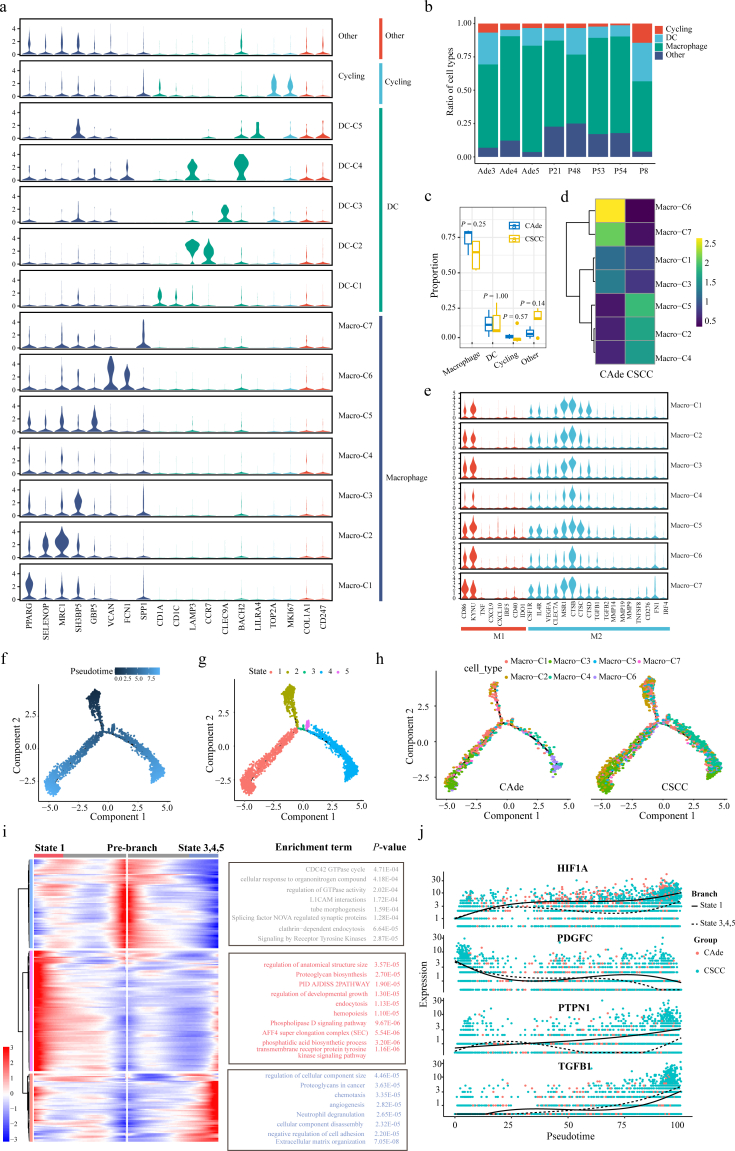
Characteristics of myeloid cells in CSCC and CAde. (a) Violin plots displayed the gene expression profiles of selected marker genes in each identified subcluster. **(b)** Bar plot displayed the proportion of each medullary subcluster in individual samples. **(c)** Box plot depicted the composition comparison for each subcluster in CSCC and CAde. The *P*-values were calculated by Wilcoxon Rank Sum Test. **(d)** Tissue preference of each macrophage subcluster revealed by R_o/e_ (ratio of observed cell number to expected cell number). **(e)** Violin plots displayed the expression profiles of M1 and M2 gene signature in each identified macrophage subcluster. **(f–h)** Developmental trajectory of macrophage cells constructed by monocle 2, colored by pseudo-time (left), state (right) and cell subtype split by pathologic type. **(i)** Pseudo-time heatmap displayed the various genes involved in the developmental process of macrophages (left), and the enrichment GO terms of gene sets of different states were also displayed (right). **(j)** Scatter plots depicted the expression levels of some representative genes during pseudo-time. The fit curves represented the gene expression of two branches, and pathologic types were denoted by color.

Myeloid cells also play important roles in the tumor microenvironment. In our study, a total of 6267 myeloid cells were re-clustered into 14 subclusters based on their marker genes, including seven macrophages (Macro-C1: PPARG; Macro-C2: SELENOP and MRC1; Macro-C3: SH3BP5; Macro-C5: GBP5; Macro-C6: VCAN and FCN1; Macro-C7: SPP1), five dendrite cells (DC-C1: CD1A; DC-C2: LAMP3 and CCR7; DC-C3: CLEC9A; DC-C4: BACH2; DC-C5: LILRA4), one cycling cell (TOP2A and MKI67), and the unknown subtype (Fig. 2i and Supplementary Figure S3a). The identified DEGs of each macrophage cell cluster were displayed in Supplementary Table S11 (|Log2FC| > 0.25, *P* < 0.05, Wilcoxon Rank Sum Test). Notably, no distinctive marker genes were identified in Macro-C4 due to its low gene expression levels. Macrophages were the major myeloid cells in each sample, and their proportion in CAde was higher compared to that in CSCC (Supplementary Figure S3b and c). The fractions of seven macrophage subclusters in each sample varied greatly (Fig. 2j). Among these subtypes, Macro-C6/7 were predominant in CAde, while Macro-C2/4/5 were more abundant in CSCC (Supplementary Figure S3d). Functional enrichment analysis revealed that Macro-C1 was involved in lipid catabolic process and sterol or cholesterol transport, which played key roles in regulating the phagocytosis and inflammatory factors of macrophages.^42^ Macro-C4 was mainly related to protein localization and cell–cell adhesion. Other clusters shared some common functional features, e.g., leucocytes and T cells activations. However, the related pathways of antigen processing and presentation were remarkably enriched in Macro-C2, which were subsequent reactions of phagocytosis and played pivotal roles in immune regulation (Fig. 2k).^43^ To further dissect the heterogeneity of identified macrophage clusters, we measured the functional score based on the known gene signatures.^29^ As shown in Fig. 2l, all these macrophage clusters could not be well separated based on the estimated scores of M1, M2, phagocytosis, angiogenesis, and myeloid-derived suppressor cells (MDSC). Each macrophage cluster shared a similar expression pattern of M1/M2-related genes (Supplementary Figure S3e). Noteworthy, Macro-C2 obtained the highest score of phagocytosis, which was consistent with its specific functions of antigen processing and presentation. Furthermore, CSCC obtained a higher phagocytosis score compared to CAde (Fig. 2m).

We used monocle2 to further explore the dynamic changes of macrophages during the transition between different subpopulations. As shown in Supplementary Figure S3f–h, all macrophages were divided into five different states, and state 2 (Macro-C1/2) was located at the starting point, while state 1 (Macro-C3/5/7) and state 3/4/5 (Macro-C4/6) were distributed in two different branches either in CSCC or CAde. DEGs involved in different states were identified (Supplementary Figure S3i), and divided into three different clusters. For example, hypoxia-inducible factor (HIF1A), growth factor (TGFB1), and inhibitory immune checkpoint (PTPN1) was gradually increased with the pseudo-time (Supplementary Figure S3j). Functional enrichment analysis revealed that cancer-related pathways (e.g., proteoglycans in cancer, chemotaxis, angiogenesis, ECM organization, and neutrophil degranulation) were active in state 3/4/5, while proteoglycan biosynthesis, endocytosis, and hemopoiesis were more active in state 1 (Supplementary Figure S3i). We further validated that Macro-C2 (MRC1^+^/CD68^+^) was significantly more abundant in CSCC compared to CAde in collected samples (Fig. 2n and o).

Collectively, the different fractions and functional status of T cells and macrophages subpopulations of these two pathological subtypes reflected their specific immune reaction to CC, which may provide insights for immunotherapy.

### Tumor-promoting vCAFs were dominant in CSCC compared to CAde

**Fig. 3:**
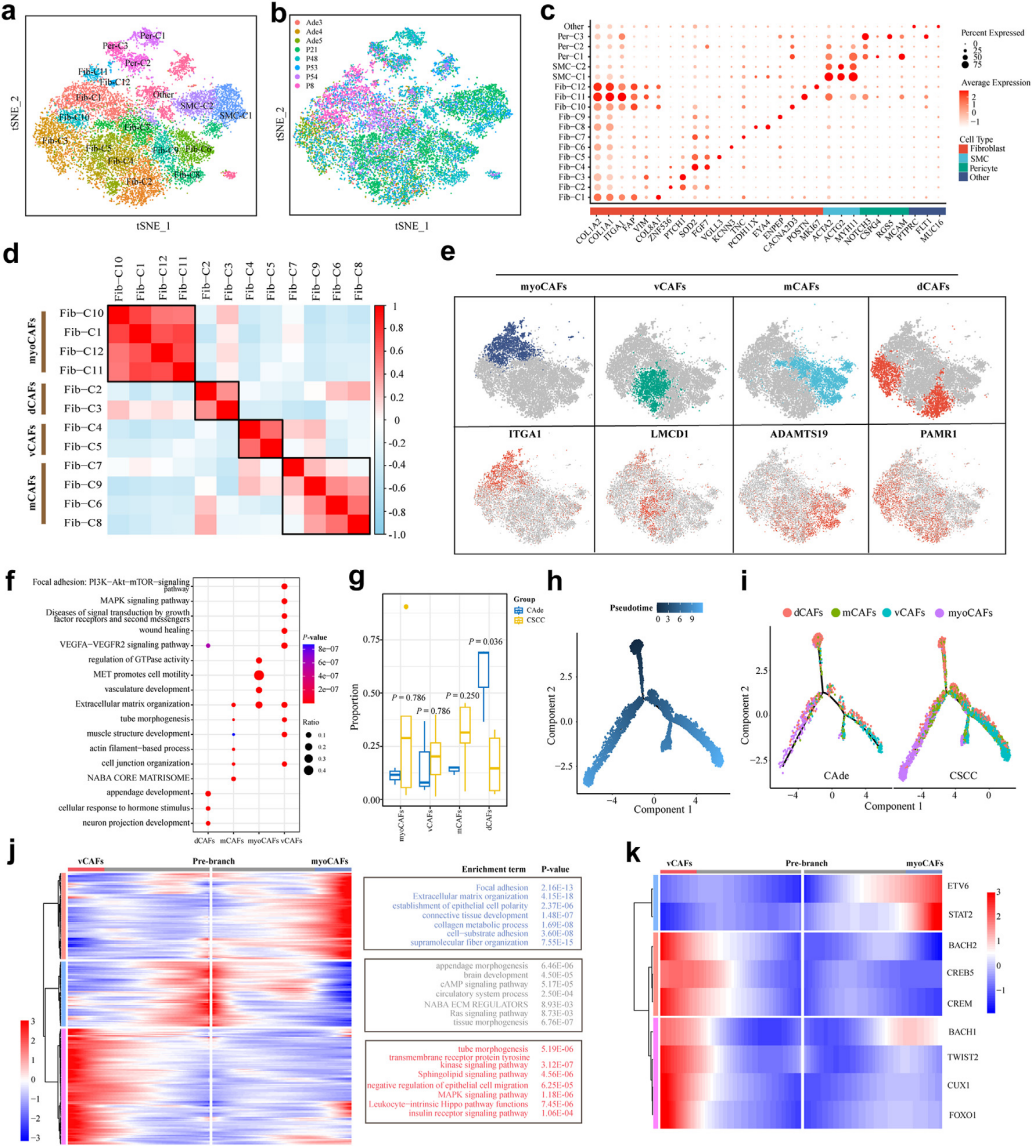
**Characteristics and subclusters of mesenchymal cells in CSCC and CAde. (a**–**b)** t-SNE plots of mesenchymal cells, colored by cell type and individual samples, respectively. **(c)** Dot plots presented the normalized expression levels of marker genes in identified mesenchymal subclusters. **(d)** Four fibroblastic subclusters were identified according to Pearson correlation coefficients based on GSEA results. **(e)** t-SNE plots of fibroblasts colored by cell types (upper) and expression of canonic genes (lower), respectively. **(f)** Dot plots displayed the GO enrichment terms of four fibroblastic subclusters. **(g)** Box plot displayed the composition comparison for four fibroblastic subclusters in CSCC and CAde. The *P*-values were calculated by Wilcoxon Rank Sum Test. **(h**–**i)** Developmental trajectory of fibroblasts constructed by monocle 2, colored by pseudo-time and pathologic types, respectively. **(j)** Pseudo-time heatmap displayed the genes involved in the developmental process of fibroblasts (left), and the enrichment GO terms of gene sets of different fibroblastic subclusters were also displayed (right). **(k)** Pseudo-time heatmap displayed dynamic changes of some representative TFs involved in the developmental process of fibroblasts.

**Supplementary Figure S4:**
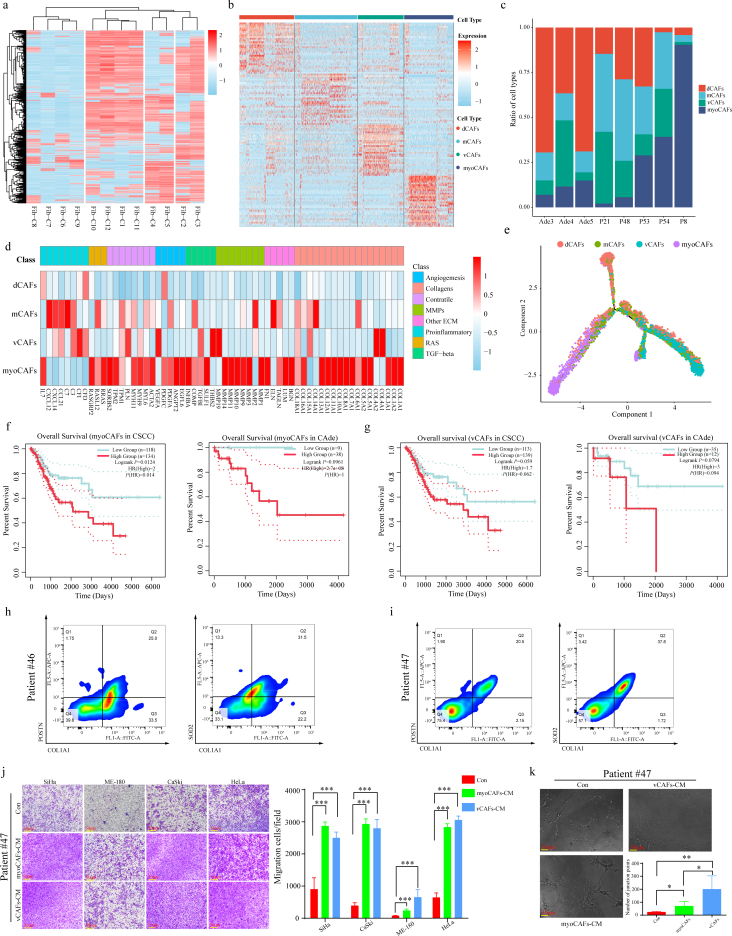
Characteristics of fibroblasts in CSCC and CAde. (a) Heatmap displayed the normalized enrichment scores (NES) of enriched terms in fibroblastic subclusters based on GSEA analysis. **(b)** Heatmap presented the expression levels of markers genes in each fibroblastic subcluster identified in fibroblast. **(c)** Bar plots displayed the proportions of each fibroblastic subcluster in individual samples. **(d)** Heatmap displayed the expression profiles of some functional gene set in fibroblastic subclusters. **(e)** Developmental trajectory of fibroblastic subclusters constructed by monocle 2, colored by subclusters. (**f**) Kaplan–Meier curves regarding the overall survival of myoCAFs based on their gene signature in CSCC cohort (left) and CAde cohort (right) from the TCGA cohort. The *P*-values were calculated by Log-rank test. (**g**) Kaplan–Meier curves regarding the overall survival of vCAFs based on their gene signature in CSCC cohort (left) and CAde cohort (right) from the TCGA database. The *P*-values were calculated by Log-rank test. (**h**) Flow cytometry plots of myoCAFs (left: POSTN^+^/COL1A1^+^) and vCAFs (right: SOD2^+^/COL1A1^+^) from one CC sample (Patient #46). (**i**) Flow cytometry plots of myoCAFs (left: POSTN^+^/COL1A1^+^) and vCAFs (right: SOD2^+^/COL1A1^+^) from another CC sample (Patient #47). (**j**) Transwell migration assay of CC cells treated with different condition medium from primary myoCAFs and vCAFs, respectively. Transwell assay had three independent biological replicates. ∗∗∗*P* < 0.001. The *P*-values were calculated by the Wilcoxon Rank Sum Test. (**k**) Blood vessel formation assay of HUVEC treated with different condition medium from primary myoCAFs, vCAFs or Con group. ∗*P* < 0.05, ∗∗*P* < 0.01. Blood vessel formation assay had three independent biological replicates. The *P*-values were calculated by the Wilcoxon Rank Sum Test.

We further re-clustered the mesenchymal cells, and four major cell types were identified, including 12 fibroblast subtypes (COL1A1 and COL1A2), two smooth muscle cell subtypes (ACTA2, ACTG2, and MYH11), three pericyte subtypes (NOTCH3 and RGS5) and another undefined fibroblast (PTPRC and MUC16) (Fig. 3a–c). To further investigate the different functional phenotypes of fibroblasts based on pathway activation and gene expression profile, 12 clusters of fibroblasts were classified into four major phenotypes using hierarchical clustering (Fig. 3d and Supplementary Figure S4a). Among these subtypes, ITGA1^+^ fibroblasts consisted of Fib-C1/C10/C11/C12 clusters were defined as myoCAFs with high expression of ITGA1, POSTN, MMP11, FN1, FAP, COL1A1 and COL8A1.^19^^,^^44^, ^45^, ^46^ ADAMTS19^+^ fibroblasts consisted of Fib-C2/C3 with high expression of ADAMTS19, NCAM1, COL14A1. LMCD1^+^ fibroblasts consisted of Fib-C4/C5 with high expression of LMCD1, EPAS1, VEGFA, COL4A1, and SOD2. PAMR1^+^ fibroblasts consisted of Fib-C6/C7/C8/C9 with high expression of PTCH1 and DACH1 (Fig. 3e and Supplementary Figure S4b). The identified DEGs of four fibroblast cell clusters were displayed in Supplementary Table S12 (|Log2FC| > 0.25, *P* < 0.05, Wilcoxon Rank Sum Test). Based on the reported classifications of fibroblasts, GO annotation revealed that PAMR1^+^ fibroblasts got involved in organ development and cellular response to hormone stimulus, which were defined as developmental CAFs (dCAFs).^47^ LMCD1^+^ fibroblasts were involved in VEGFA-VEGFR2 signaling pathway, wound healing, tube morphogenesis, MAPK, and PI3K-Akt-mTOR signaling pathway, which were further defined as vCAFs.^47^, ^48^, ^49^ ADAMTS19^+^fibroblasts mainly got involved in NABA CORE MATERISOME and ECM organization, which were defined as matrix CAFs (mCAFs).^50^ myoCAFs mainly mediated the pathway of extracellular matrix (ECM) organization and MET promotes cell motility (Fig. 3f). Fractions of each fibroblastic subtype also varied greatly in different samples (Supplementary Figure S4c), myoCAFs, mCAFs and vCAFs seemed to be more abundant in CSCC, although *P*-values was not statistically significant. While the proportion of dCAFs in CAde samples was significantly higher compared to that in CSCC samples (*P* *=* 0.036, Wilcoxon Rank Sum Test) (Fig. 3g). The expression profiles of some known functional signatures (angiogenesis, collagens, TGF-beta, etc.) in identified fibroblastic subtypes were also measured (Supplementary Figure S4d).

Next, monocle2 was employed to infer the potential developmental trajectory of fibroblast. Interestingly, we found mCAFs and dCAFs were mainly located at the beginning point of the developmental trajectory, while myoCAFs and vCAFs were located at two branches separately (Fig. 3h and Supplementary Figure S4e). The same developmental trajectories of fibroblasts were observed in CSCC and CAde (Fig. 3i). Moreover, gene expression profiles were further clustered into three different clusters during the developmental process, and further functional enrichment analysis revealed that cancer-related pathways (including MAPK signaling pathway, and insulin receptor signaling pathway) were active during the process of vCAFs transition. While ECM organization, connective tissue development, and collagen metabolic process were active during the process of myoCAFs transition (Fig. 3j). To further explore the regulatory mechanism during the pseudo-time, abnormally active TFs were identified. Some metastasis-related TFs (e.g., BACH2, TWIST2, CREB5, and CUX1) and pro-angiogenic TFs (FOXO1, BACH1) were involved in the vCAFs developmental process, while ETV6 and STAT2 were involved in the myoCAFs developmental process (Fig. 3k).^51^, ^52^, ^53^, ^54^, ^55^

**Fig. 4:**
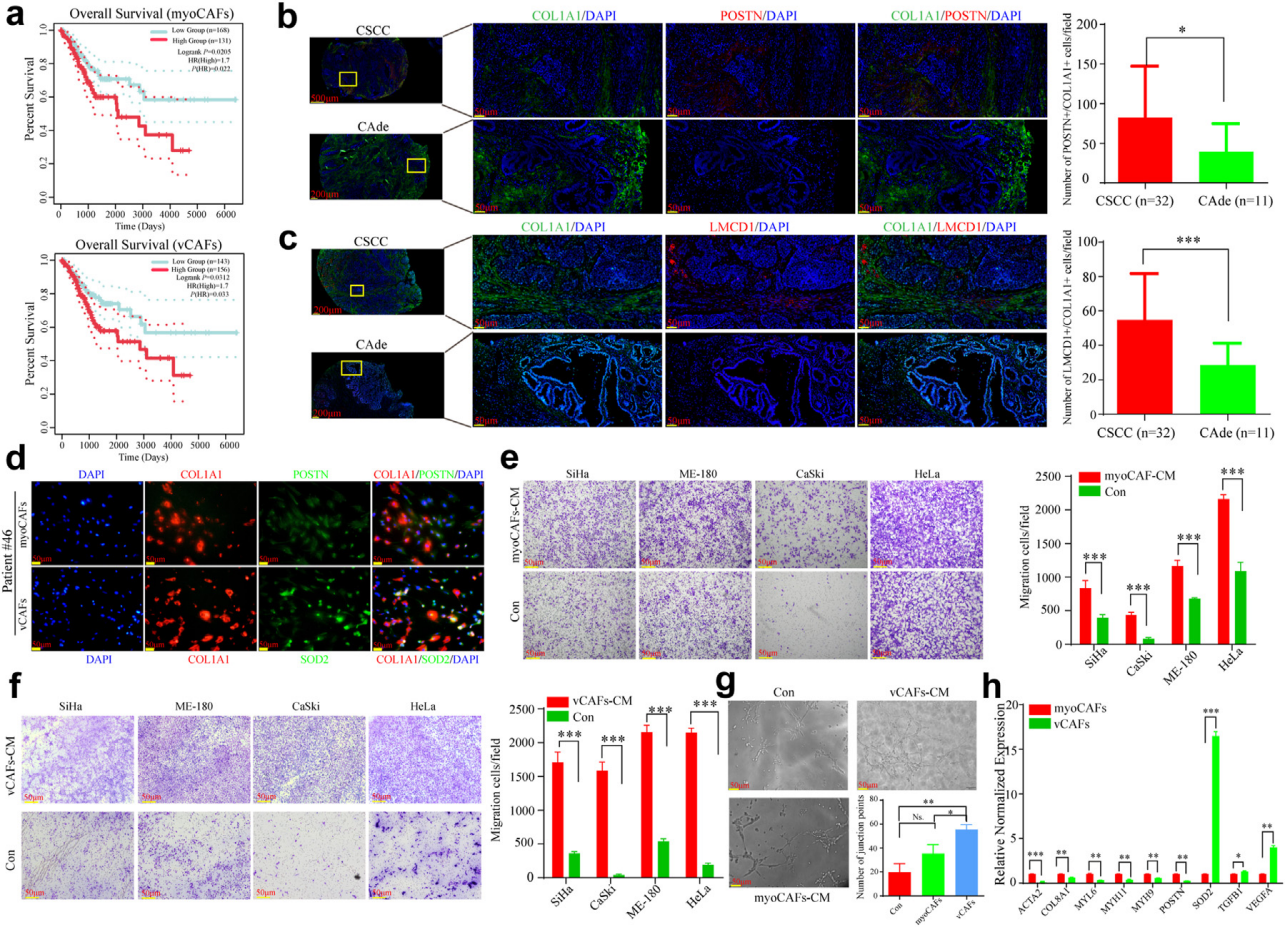
**Functional validation of identified pro-tumoral fibroblasts. (a)** Kaplan–Meier curves regarding the overall survival of myoCAFs (upper) and vCAFs (lower) based on their gene signature in all CC cohort from the TCGA database. *P*-value was calculated by Log-rank test. **(b**–**c)** mIF staining of myoCAFs (upper: POSTN^+^/COL1A1^+^) and vCAFs (lower: LMCD1^+^/COL1A1^+^) in collected CSCC (n = 32) and CAde (n = 11) samples. ∗*P* < 0.05, ∗∗∗*P* < 0.001. The *P*-values were calculated by Wilcoxon Rank Sum Test. **(d)** Immunofluorescence staining of myoCAFs (upper: POSTN^+^/COL1A1^+^) and vCAFs (lower: SOD2^+^/COL1A1^+^) sorted by flow cytometry from one clinical CC sample. **(e**–**f)** Transwell migration assay of CC cells treated with different condition medium from primary myoCAFs and vCAFs, respectively. Transwell assay had three independent biological replicates. ∗∗∗*P* < 0.001. The *P*-values were calculated by Wilcoxon Rank Sum Test. **(g)** Blood vessel formation assay of HUVEC treated with different condition medium from primary myoCAFs, vCAFs or Con group. ∗*P* < 0.05, ∗∗*P* < 0.01, Ns not significant. Blood vessel formation assay had three independent biological replicates. The *P*-values were calculated by Wilcoxon Rank Sum Test. **(h)** mRNA expression levels of some marker genes in primary myoCAFs and vCAFs. ∗*P* < 0.05, ∗∗*P* < 0.01, ∗∗∗*P* < 0.001. Each target gene had three independent biological replicates. The *P*-values were calculated by Wilcoxon Rank Sum Test.

We then measured the prognostic significance of identified fibroblastic subtypes using the TCGA cohorts. myoCAFs were negatively correlated with patient overall survival in all CC cohort (Log-rank *P* = 0.0205, Log-rank test), CSCC cohort (Log-rank *P* = 0.0124, Log-rank test) from the TCGA database, but not in the CAde cohort (Log-rank *P* = 0.0961, Log-rank test) (Fig. 4a and Supplementary Figure S4f). vCAFs were also negatively correlated with patient overall survival in all CC cohort (Log-rank *P* = 0.0312, Log-rank test) from the TCGA database, but not in the CSCC cohort (Log-rank *P* = 0.059, Log-rank test) and the CAde cohort (Log-rank *P* = 0.0794, Log-rank test) (Fig. 4a and Supplementary Figure S4g). We further validated that vCAFs and myoCAFs were more abundant in CSCC compared to CAde with external samples using mIF (Fig. 4b and c). We then extracted and cultured primary fibroblasts from one collected CC sample (Patient #46). Primary myoCAFs (POSTN^+^/COL1A1^+^) and vCAFs (SOD2^+^/COL1A1^+^) were sorted by flow cytometry (Supplementary Figure S4h), and further validated them by immunofluorescence staining (Fig. 4d). Transwell assay revealed that the CM of myoCAFs and vCAFs all could remarkably promote migrative abilities of CC cells (Fig. 4e and f). CM of vCAFs seemed to display higher capacities of promoting angiogenesis compared to the CM of myoCAFs or Control group (Fig. 4g). Furthermore, the expression levels of TGFB1 and VEGFA in vCAFs were significantly higher than those in myoCAFs, which were proved to promote tumor progression and angiogenesis (Fig. 4h).^54^^,^^56^ Also, some up-regulated genes in myoCAFs (e.g., ACTA2, COL8A1, MYL6, MYH11, MYH9, POSTN) were further validated by qRT-PCR, which were consistent with the findings in Supplementary Figure S4d. We also extracted and cultured primary fibroblasts from another CC sample (Patient #47) (Supplementary Figure S4i). Further experimental validation was performed, and we also found that the CM of myoCAFs and vCAFs from this patient could remarkably promote migrative abilities of CC cells (Supplementary Figure S4j). CM of vCAFs and myoCAFs also displayed high capacities of promoting angiogenesis (Supplementary Figure S4k). Collectively, our findings confirmed that primary vCAFs and myoCAFs from CC samples displayed pro-metastatic potential *in vitro*.

### Prognosis-related lymphatic endothelial cells (LEC) were enriched in CSCC

**Supplementary Figure S5:**
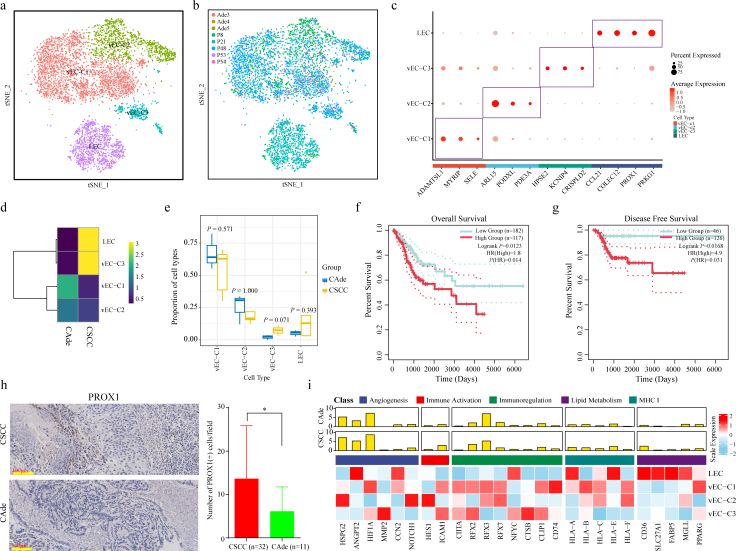
Characteristics and subclusters of endothelial cells (ECs) in CSCC and CAde. (a-b) t-SNE plots of ECs, colored by cell types and individual samples, respectively. **(c)** Dot plots presented the normalized expression levels of marker genes in each EC subcluster. **(d)** Tissue preference of identified EC subcluster revealed by R_o/e_ (ratio of observed cell number to expected cell number). **(e)** The box plot presented the composition comparison for each subcluster of ECs in CSCC and CAde. The *P*-values were calculated by Wilcoxon Rank Sum Test. **(f–g)** Kaplan–Meier curves of overall survival (left) and disease-free survival (right) based on the gene signature of lymphatic endothelial cells (LECs). The *P*-value was calculated by Log-rank test. **(h)** IHC staining of LECs (PROX1) in collected CSCC (n=32) and CAde (n=11) samples. ∗ *P* < 0.05. The *P*-value was calculated by Wilcoxon Rank Sum Test. **(i)** Heatmap displayed the functional activities of identified EC subclusters based on known gene signatures. The upper panel depicted the average expression levels of representative genes in pathologic types, and the lower panel depicted the average expression levels of representative genes in each identified cell subcluster.

Endothelial cell (EC) is a major component with vast heterogeneity of tumor microenvironment by promoting tumor growth and metastasis.^57^, ^58^, ^59^ To further explore the differences of ECs between CSCC and CAde, 6040 ECs were divided into three vascular endothelial cells (vEC) subclusters (vEC-cluster 1: ADAMTSL1 and MYRIP, vEC-cluster 2: PODXL and ARL15, vEC-cluster 3: HPSE2 and KCNIP4) andone LEC subcluster (CCL21 and PROX1) based on marker gene expression profiles (Supplementary Figure S5a–c). Fraction analysis of each subcluster revealed that vEC-cluster 3 and LEC were more abundant in CSCC compared to CAde, while vEC-cluster 1 and vEC-cluster 2 were enriched in CAde (Supplementary Figure S5d and e). Prognosis analysis revealed that LEC significantly correlated with patients OS (Log-rank *P* = 0.0123, Log-rank test) and disease-free survival (Log-rank *P* = 0.0168, Log-rank test), but not with vEC-cluster 1/2/3 (Supplementary Figure S5f and g). We then confirmed that LEC was indeed more abundant in CSCC samples compared to CAde samples using IHC (Supplementary Figure S5h). LEC has been proven to be crucial for distant metastasis or self-renewal and development of cancer stem cells in other malignancies^60^^,^^61^ To explain why LEC showed negative prognostic effects on CC, we measured the expression of some known gene signatures in four identified subtypes.^59^^,^^62^ As shown in Supplementary Figure S5i, the related genes of angiogenesis-related genes (ANGPT2 and CCN2) and lipid metabolism (CD36, SLC27A1, FABP5, and MGLL) were up-regulated in LEC subtype, while the related genes of immune activation (HES1 and ICAM1) and immunoregulation (CIITA, RFX2, RFX3, RFX7, CTSB, CLIP1, and CD74) were down-regulated in LEC subtypes. Collectively, the deficient immunosurveillance of LEC could explain the negative impact of LEC abundance on CC prognosis.

### Transcriptional heterogeneity of malignant epithelial cells between CSCC and CAde

#### HPV infection affects malignant epithelial cells’ stemness and cell cycle

**Supplementary Figure S6:**
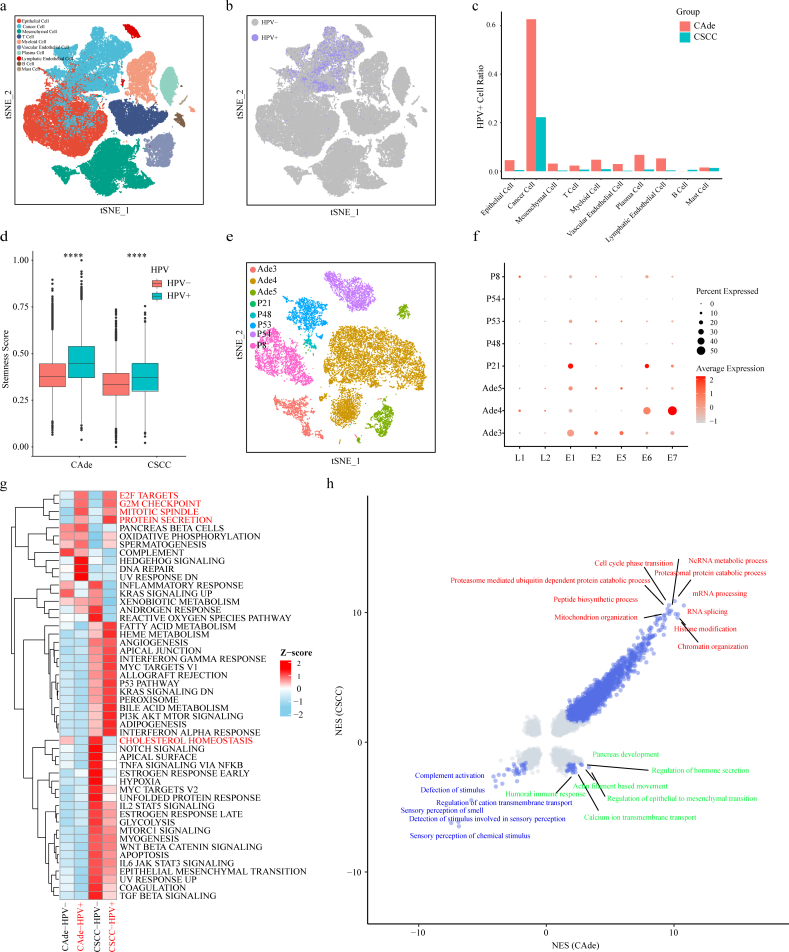
The different characteristics of HPV infection in CSCC and CAde. (a-b) t-SNE plots showed the 10 cell types and their corresponding HPV infection status. **(c)** The bar plot displayed the ratio of HPV-positive (HPV^+^) in each cell type. **(d)** Box plots displayed the estimated stemness scores of cancer cells with different HPV infection statues in CSCC and CAde. ∗∗∗∗*P* < 0.0001. The *P*-values were calculated by Wilcoxon Rank Sum Test. **(e)** t-SNE plot depicted the distribution of HPV^+^ cancer cells in eight individual samples. **(f)** Dot plots displayed the expression profiles of HPV-related genes in eight CC samples. **(g)** Heatmap depicted the z-score of hallmark pathways in cancer cells with different HPV infection statues. **(h)** Scatter plots displayed the enrichment scores (NES) of enriched terms in CSCC and CAde. The blue dots represented the significant enrichment terms (MSigDB_C5_BP, *P*-value < 0.05), while the grey dots represented no significance. The label colors corresponded to the specific enrichments of HPV^+^ status in CSCC and CAde (red), HPV^-^ status (blue) in CSCC and CAde, and HPV^+^ status in CAde (green).

CC has definitely pathogenic factor, that is, persistent HPV infection. Previous studies have well illustrated the molecular pathogenesis of HPV infection, but the biological differences between HPV-negative and HPV-positive malignant epithelial cells at the single-cell resolution remain unknown. Here, we measured the expression of HPV-related genes (L1, L2, E1, E2, E5, E6, and E7) in all identified cell types, and classified cells with the expression of any HPV-related gene as HPV-positive cells, and vice-versa. As shown in Supplementary Figure S6a–c, the HPV-positive rate of cancer cells was the highest, whether in CSCC or CAde samples. Here, we found that the estimated cancer stemness score of HPV-positive cells was significantly higher than that of HPV-negative cells in both CSCC and CAde samples (Supplementary Figure S6d), which indicated that HPV infection may reshape the tumor microenvironment by enhancing cancer stemness. After re-clustering HPV-positive cancer cells according to the source of patients, we found that E6, E7, and E1 displayed higher expression compared to other HPV-related genes (Supplementary Figure S6e and f). Furthermore, Ade-4 sample exhibited the most abundant HPV-related genes, which indicated the vast intra- and inter-heterogeneity of HPV-related gene expression. Further functional score analysis on cancer cells with different HPV-statues revealed that the cell cycle-related pathways (E2F TARGETS, G2M CHECKPOINT, and MITOTIC SPINDLE) were active after HPV infection both in CSCC and CAde. However, the activity of CHOLESTEROL HOMEOSTASIS, which was related to carcinogenesis and tumor microenvironment remodeling was reduced (Supplementary Figure S6g).^63^^,^^64^ There were some other different dysregulated pathways between CSCC and CAde samples after HPV infection. For example, the pathways of INTERFERON GAMMA RESPONSE and INTERFERON ALPHA RESPONSE were up-regulated in HPV-positive cancer cells of CSCC, but not in CAde. Similar findings were observed using the Gene Set Enrichment Analysis (GSEA) (Supplementary Figure S6h). Collectively, our study revealed the external and internal tumor features caused by HPV infection, which may deepen our understanding of HPV carcinogenicity.

#### Distinctive malignant epithelial subtypes and differentially expressed genes between CSCC and CAde

**Fig. 5:**
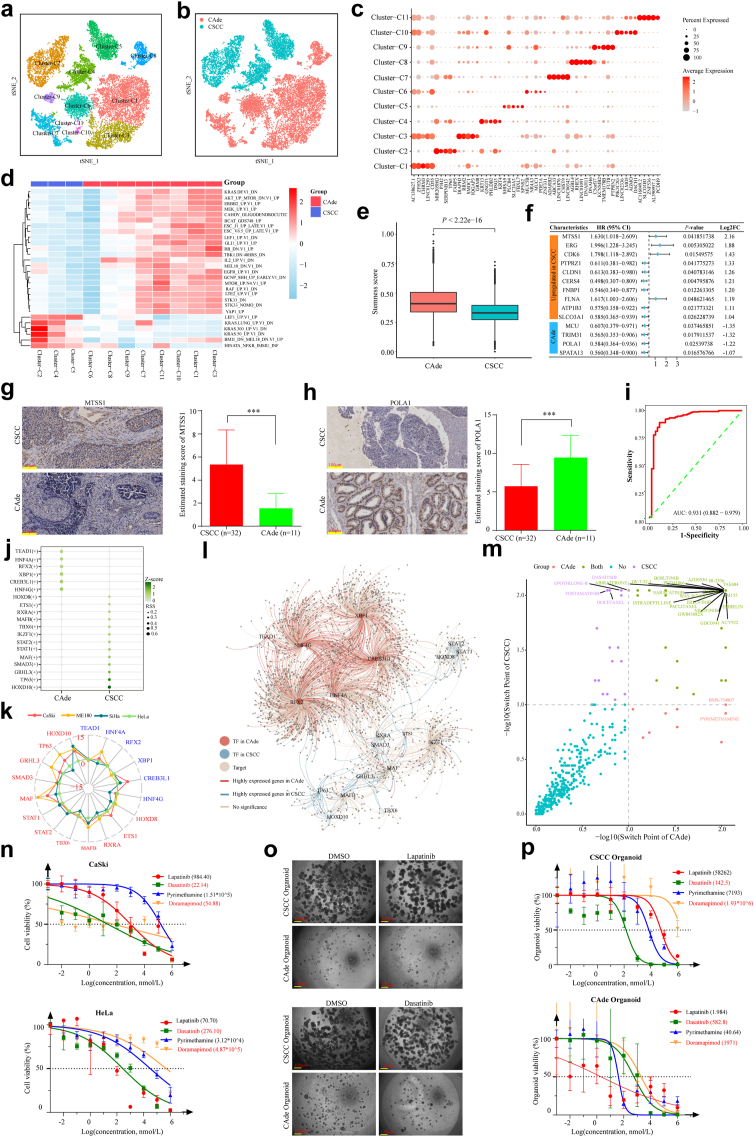
**The transcriptomic heterogeneities of malignant cells in CSCC and CAde. (a**–**b)** t-SNE plots of malign epithelial cells, colored by cell clusters and individual samples, respectively. **(c)** Dot plots revealed the gene expression profiles of each malignant cell cluster. **(d)** Heatmap revealed the molecular functions of each malignant cell cluster based on GSVA analysis (MSigDB_C6). **(e)** The box plot depicted the stemness score of malignant cells in CSCC and CAde. The *P*-value was calculated by Wilcoxon Rank Sum Test. **(f)** Forest plot of the identified differentially expressed genes from snRNA-seq data for CSCC and CAde regarding overall survival. **(g)** Representative IHC staining of MTSS1 in collected CSCC (n = 32) and CAde (n = 11) samples. ∗∗∗*P* < 0.001. The *P*-value was calculated by Wilcoxon Rank Sum Test. **(h)** Representative IHC staining of POLA1 in collected CSCC (n = 32) and CAde (n = 11) samples. ∗∗∗*P* < 0.001. The *P*-value was calculated by Wilcoxon Rank Sum Test. **(i)** The ROC curve (sensitivity against 1-specificity) of identified specific gene signature for classifying CSCC and CAde based on TCGA CESC cohort. **(j)** Dot plots displayed the abnormally activated transcription factors (TFs) for CSCC and CAde. **(k)** Radar plot displayed the expression profiles of identified TFs in CC cell lines. The distance from the dots to the center of the circle represented the log2 (relative gene expression). **(l)** Gene regulatory network among TFs and target genes in malignant epithelial cells. Significant genes meant the adjusted *P*-value <0.01 and |Log2FC| > 0.25. *P*-value was calculated by Wilcoxon Rank Sum t-test. **(m)** Scatter plots of switch point of a certain drugs in CSCC and CAde. The switch point of certain drug less than 0.1 was defined as sensitivity, and the sensitivity of drugs in CSCC and CAde were denoted by color. The Switch Point value of 0 was converted to 0.009 for plotting. **(n)** IC50 values of selected drugs (Pyrimethamine, Lapatinib, Dasatinib and Doramapimod) in CaSki and HeLa cell line. Each drug had at least three biological replicates in each cell line. **(o)** Morphological changes of constructed organoids from CSCC and CAde samples after being treated with Lapatinib and Dasatinib. **(p)** IC50 values of selected drugs (Pyrimethamine, Lapatinib, Dasatinib and Doramapimod) in CSCC- and CAde organoids. Each drug had at least three biological replicates in each constructed organoid.

**Supplementary Figure S7:**
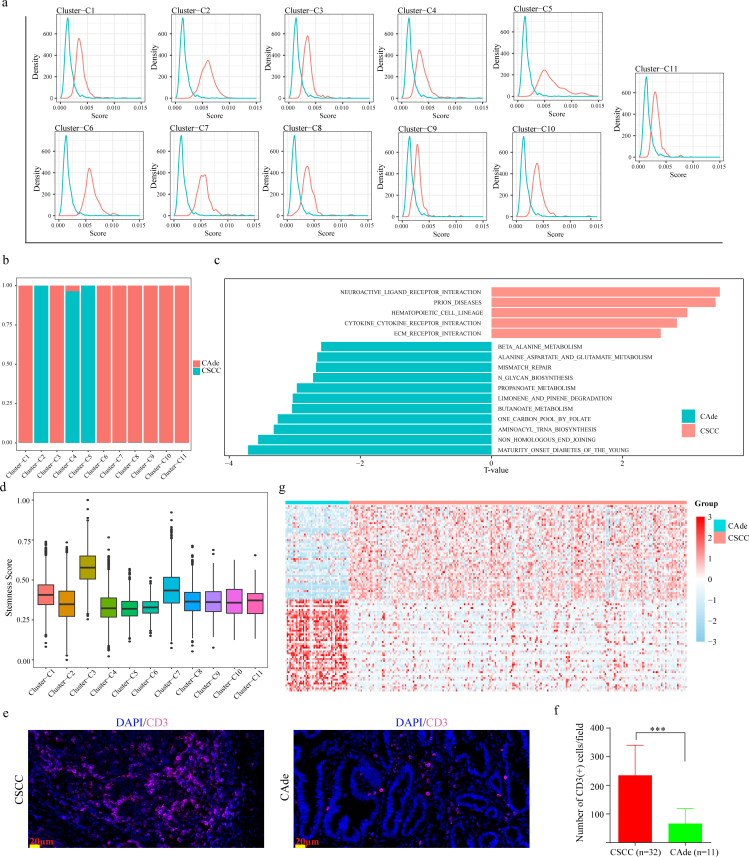
Special characteristics of malignant epithelial cells from CSCC and CAde. (a) Density plots displayed the CNV scores of subclusters against reference cells in malignant epithelial cells. **(b)** Bar plot depicted the proportions of identified malignant epithelial cells clusters in CSCC and CAde. **(c)** Enriched KEGG pathway of malignant epithelial cells from CSCC and CAde. **(d)** Box plot displayed the stemness score of each malignant epithelial cell cluster. **(e)** mIF staining of T cells (CD3^+^) in collected CSCC (n=32) and CAde (n=11) samples. **(f)** Box plots displayed the number of T cells (CD3^+^)/field in CSCC and CAde. ∗∗∗ *P* < 0.001. The *P*-value was calculated by Wilcoxon Rank Sum Test. **(g)** Heatmap displayed the expression profiles of identified differentially expressed genes from the snRNA-seq data in TCGA cohort.

To better reveal the specific tumor-intrinsic features of CSCC and CAde, we re-divided all malignant epithelial cells into 11 clusters and identified their marker genes (Fig. 5a–c), and each cluster obtained a higher CNV score compared to reference cell (Supplementary Figure S7a). Cluster-1/3/6/7/8/9/10 predominantly comprised CAde samples, while other clusters mainly composed of CSCC samples (Supplementary Figure S7b). Interestingly, malignant epithelial subpopulations derived from CSCC and CAde could be well separated based on the GSVA analysis (Fig. 5d and Supplementary Figure S7c). Remarkably, the related pathways of AKT, MTOR, and ERBB2 were active in CAde samples, while HINATA_NFKB_IMMU-INF was active in CSCC samples. These findings indicated the different intrinsic functions between CSCC and CAde, which could facilitate in revealing different pathogenesis and selecting sensitive targeted drugs for patients with CSCC and CAde.

**Supplementary Figure S8:**
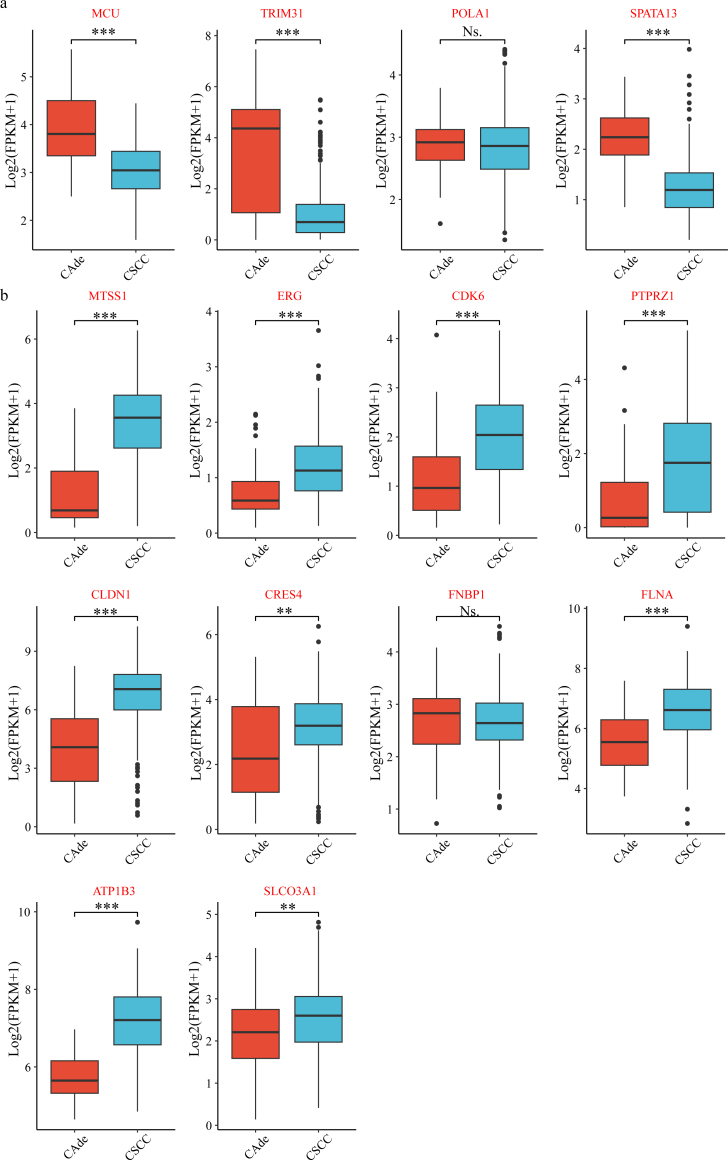
Expression profiles of some identified differentially expressed genes in TCGA CC cohort. (a) Box plots displayed the expression profiles of specific genes for CAde in TCGA cohort. ∗∗∗*P* < 0.001, Ns not significant. The *P*-values were calculated by Wilcoxon Rank Sum Test. **(b)** Box plots displayed the expression profiles of specific genes for CSCC in TCGA cohort. ∗∗*P* < 0.01, ∗∗∗*P* < 0.001, Ns not significant. The *P*-values were calculated by Wilcoxon Rank Sum Test.

Cancer stemness has been proven to affect immunosuppression in a range of human cancers by expelling T cells.^65^ We also measured the stemness score of each cluster (Supplementary Figure S7d), and found that CAde obtained higher tumor stemness compared to CSCC, which indicated that CAde had a more inhibitory immune microenvironment (Fig. 5e). We further verified this finding in the tissue microarray, mIF staining revealed that the infiltration of T (CD3^+^) cells was significantly higher in CSCC compared to that in CAde samples (Supplementary Figure S7e and f). We then identified the DEGs of cancer cells between CSCC and CAde at the single-cell resolution (|Log2FC| >0.25, *P* < 0.05, Wilcoxon Rank Sum Test) (Supplementary Table S13). It was interesting to note that the identified top 50 DEGs displayed the same expression pattern in the TCGA cohort (Supplementary Figure S7g). Based on this, we aimed to construct a gene panel that could distinguish CSCC from CAde. After resetting the threshold (|Log2FC| >1, *P* < 0.05, Wilcoxon Rank Sum Test), a gene set containing 14 prognosis-related genes was established (Fig. 5f). To evaluate the accuracy of our established discriminative gene panel, we measured their expression in TCGA cohort (Supplementary Figure S8) and performed IHC staining on a tissue microarray. As shown in Fig. 5g and h, MTSS1 was confirmed to be over-expressed in CSCC samples, while POLA1 was up-regulated in CAde samples. The receiver operating characteristic (ROC) curve confirmed this gene panel exhibited good performance in classifying CAde and CSCC (AUC = 0.93, 95% CI = 0.882–0.979) (Fig. 5i).

#### Identification of a series of chemotherapy drugs targeting CSCC and CAde

**Supplementary Figure S9:**
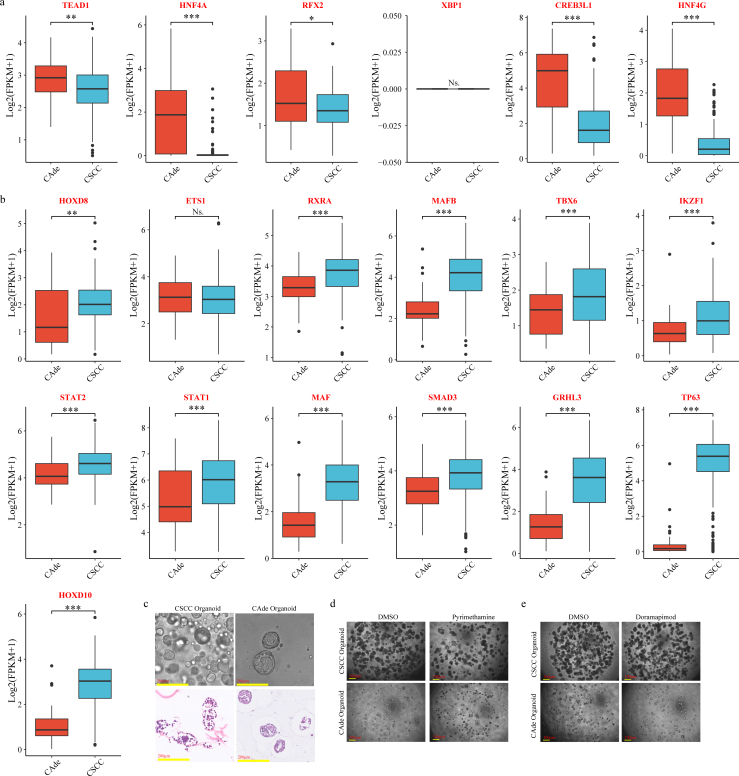
Expression profiles of some specific TFs in TCGA CC cohort. (a) Box plot displayed the expression profiles of some specific TFs for CAde in TCGA CC cohort. ∗*P <* 0.05, ∗∗*P* < 0.01, ∗∗∗*P* < 0.001. Ns not significant. The *P*-values were calculated by Wilcoxon Rank Sum Test. **(b)** Box plot displayed the expression profiles of some specific TFs for CSCC in TCGA CC cohort. ∗∗*P* < 0.01, ∗∗∗*P* < 0.001. Ns not significant. The *P*-values were calculated by Wilcoxon Rank Sum Test. **(c)** Constructions of organoids from CSCC and CAde samples. **(d)** Morphological changes of constructed organoids from CSCC and CAde samples after being treated with Pyrimethamine. **(e)** Morphological changes of constructed organoids from CSCC and CAde samples after being treated with Doramapimod. ∗*P <* 0.05, ∗∗*P* < 0.01, ∗∗∗*P* < 0.001. Ns not significant. The *P*-values were calculated by Wilcoxon Rank Sum Test.

Combined with the identified DEGs, we further dissected the heterogeneity of upstream regulatory molecules (i.e., TFs) using the single-cell regulatory network inference and clustering (SCENIC) algorithm.^66^ As shown in Fig. 5j, a series of specific TFs were identified for CSCC (HOXD8, ETS1, RXRA, MAFB, TBX6, IKZF1, STAT2, STAT1, MAF, SMAD3, GRHL3, TP63, and HOXD10) and CAde (TEAD1, HNF4A, RFX2, XBP1, CREB3L1, and HNF4G). We further measured their expression in CSCC (SiHa, CaSki, and ME-180) and CAde (HeLa) cell lines as well as TCGA cohort (Fig. 5k and Supplementary Figure S9a and b). The TF-gene regulatory networks indicated the over-expressed genes of CAde were mainly regulated by the CAde-specific TFs, and vice versa in CSCC (Fig. 5l). Then, Beyondcell (v1.3.3) algorithm was used to predict drug sensitivity based on the expression profiles of malignant cells (Fig. 5m). Details of the top 10 identified drugs with high sensitivity for CSCC and CAde were displayed in Supplementary Table S14. We randomly selected some drugs to further validate the prediction accuracy of our drug screening. As shown in Fig. 5n, the IC50 values of CAde-specific drugs (dihydrofolate reductase inhibitor: Pyrimethamine; ERBB2 inhibitor: Lapatinib) in HeLa were smaller compared to that in CaSki. In contrast, the CSCC-specific drugs (STAT5 inhibitor: Dasatinib; MAPK inhibitor: Doramapimod) had a better cytotoxic function in CaSki. We also constructed organoids from clinical CSCC and CAde samples to better test drug sensitivity (Supplementary Figure S9c), and the results were basically consistent with those in CC cell lines (Fig. 5o and p and Supplementary Figure S9d and e). These findings indicated the strong reliability of our identified drugs, which were worth further exploration in future.

Collectively, we distinguished specific malignant epithelial subpopulations between the two subtypes of CC and established a discriminative gene panel based on their DEGs. Furthermore, we predicted and verified some specific chemotherapy drugs for CSCC and CAde, which could provide insights for exploring individualized treatments for both pathologic CC in clinical practice.

### Specific cellular interactions between cancer cells and fibroblasts in CSCC and CAde

**Fig. 6:**
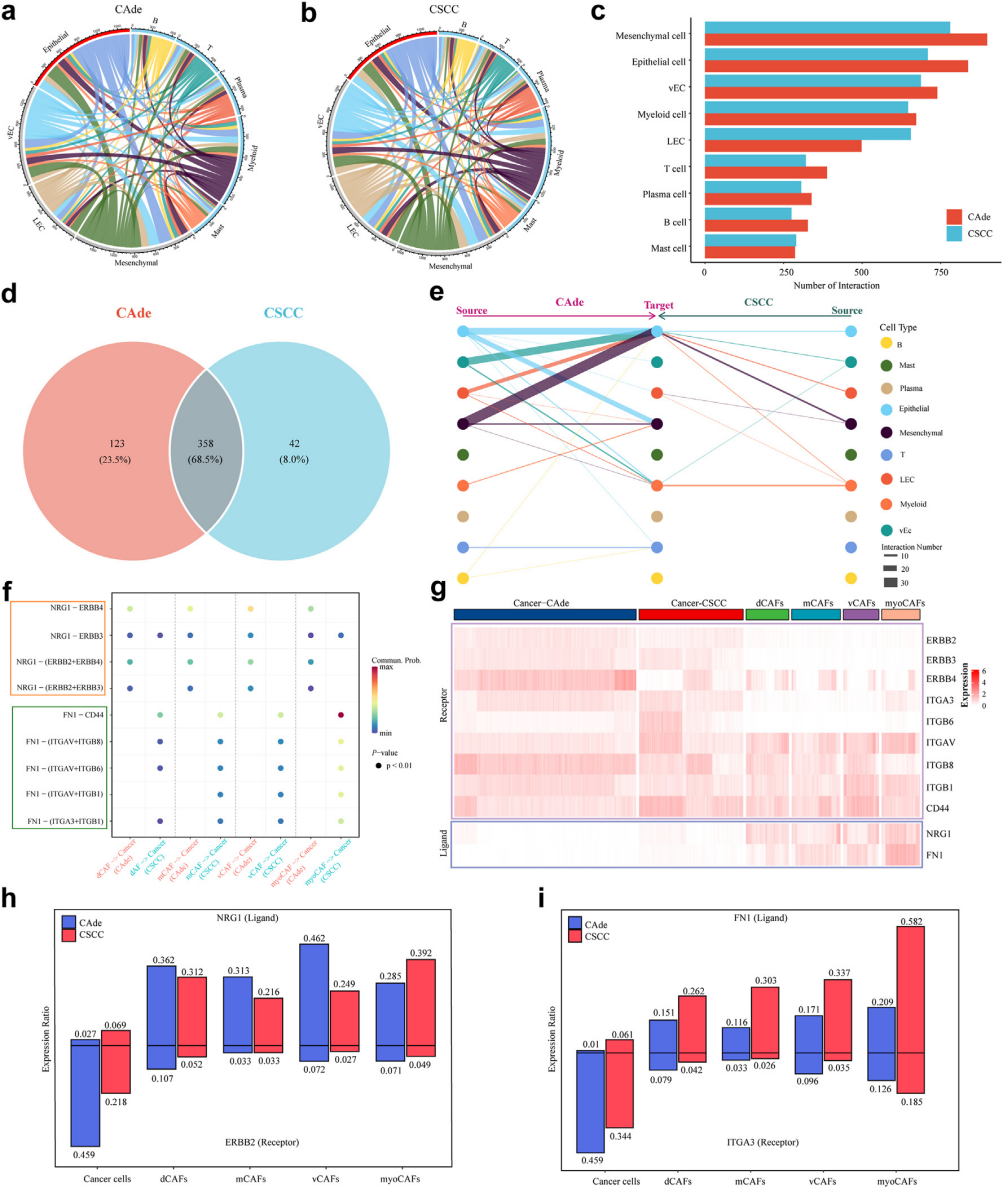
**Specific communication patterns between malignant cells and fibroblasts in CSCC and CAde. (a**–**b)** The landscape of cell communications among major cell types in CAde and CSCC, respectively. The color of lines represented major cell types. **(c)** Bar plot displayed the number of significant interactions among the major cell types of CSCC and CAde. **(d)** Venn plot displayed the numbers of specific interactions in CSCC and CAde. **(e)** Hierarchical diagram displayed the communication possibility of selected interaction pairs among source cells and target cells in CSCC and CAde. **(f)** Bubble plots displayed the communication probability of selected interaction pairs between cancer cells and fibroblasts in CSCC and CAde. **(g)** Heatmap displayed the expression levels of specific receptors and ligands in cancer cells and identified CAFs. **(h)** The expression ratios of NRG1-ERBB2 interaction in cancer cells and CAFs. **(i)** The expression ratios of FN1-ITGA3 interaction in cancer cells and CAFs.

To investigate the heterogeneity of cell–cell interactions between CSCC and CAde, a set of ligand-receptor (L-R) pairs from CellphoneDB database were used to predict potential interactions among nine identified major cell types. CAde had more abundant interaction pairs compared to those in CSCC, especially in epithelial and mesenchymal cells (Fig. 6a–c). Then we identified the specific ligand-receptor pairs that belonged to CSCC and CAde. Interestingly, we found that there were more specific ligand-receptor pairs in CAde than in CSCC (Fig. 6d). About 68.5% significant interaction pairs were shared by CAde and CSCC, while 23.5% interaction pairs were specific for CAde. Moreover, epithelial cells dominated specific signaling from mesenchymal cells both in CSCC and CAde, which was the major focus of our following analysis (Fig. 6e). Further CellChat analysis revealed that the signaling pathways between cancer cells and identified CAFs (NRG1-ERBB2/4 and FN1-CD44/ITGAV/ITGA3/ITGB6/ITGB8/ITGB1) were specifically enriched in CAde and CSCC, respectively (Fig. 6f). Remarkably, some receptors (ERBB2, ITGA3, and ITGB6) were mainly expressed in cancer cells, and their putative ligands (NRG1 and FN1) were over-expressed in CAFs (Fig. 6g). Moreover, the interdependent ligand-receptor pairs of NRG1-ERBB2 and FN1-ITGA3 displayed the prominent expression in cellular components of CAde and CSCC, respectively (Fig. 6h and i).

Interestingly, the identified Lapatinib, an ERBB2 inhibitor, was particularly sensitive to CAde samples. Previous studies have proven that STAT5 and MAPK were pivotal downstream molecules of integrin in malignant tumors, and we found that the identified Dasatinib and Doramapimod, targeting STAT5 and MAPK molecules, displayed specific sensitivities to cancer cell line and organoids of CSCC.^67^^,^^68^ Collectively, we identified specific cellular interactions for CSCC (FN1-ITGA3) and CAde (NRG1-ERBB2) between CAFs and cancer cells, which could also provide insights into selecting promising therapeutic chemotherapy drugs for these two different CC types.

## Discussion

**Fig. 7:**
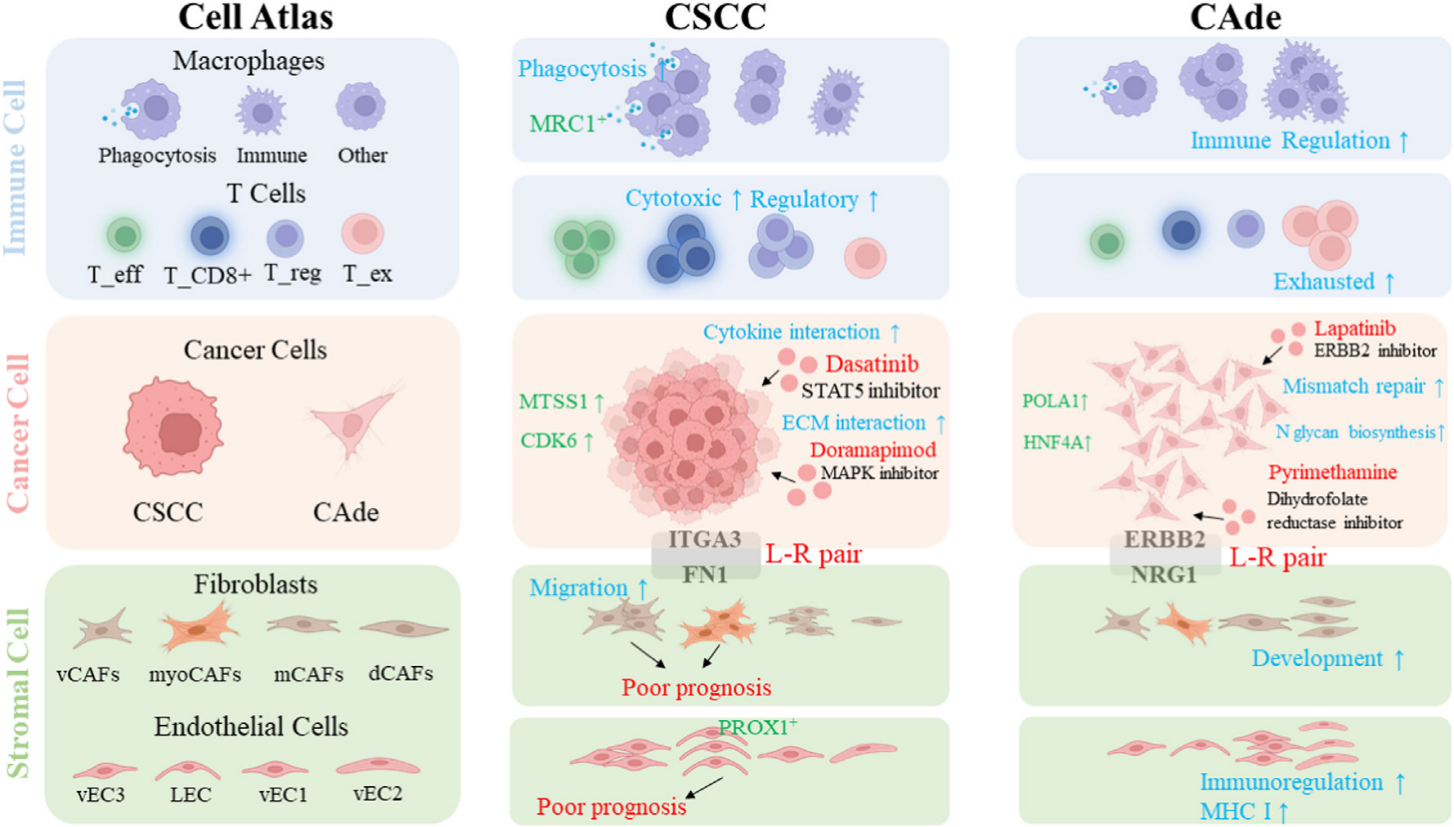
Main content analysis and findings of this study.

Here, we depicted different transcriptomic atlas of five CSCC and three CAde samples at the single-cell solution. The heterogeneities between these two pathological types regarding epithelial cells, immunocytes and fibroblasts have been well documented, and some interesting findings (e.g., specific chemotherapy drugs, identified LECs, identified myoAFs or vCAFs) were further validated by mIF staining, extracting primary CAFs, constructing CC-organoids, etc (Fig. 7). Compared to previous studies on CC by single-cell RNA sequencing, our study provided a valuable resource for revealing the vast heterogeneity of carcinogenesis and tumor microenvironment between CSCC and CAde, and for designing personalized treatments strategies in the future.

Cancer stem cells (CSCs) with the capacity for self-renewal and diverse differentiation, have been proven to play critical roles in carcinogenesis, development, metastasis, recurrence, and treatment resistance of malignant tumors.^69^ As an HPV-induced tumor, the effect of HPV infection on the stemness of epithelial cells in CC remains inconclusive. Zhang et al. revealed that HPV16 infection elevated intrinsic stemness of head and neck squamous cell carcinoma by up-regulating ALDH1.^70^ The E6 and E7 oncoproteins were reported to enhance the stemness of CC cells by up-regulating HES1 and APH1B, respectively.^71^^,^^72^ Furthermore, our study confirmed that HPV infection could elevate stemness score of malignant epithelial cells, and the HPV positivity rate and the stemness score of cancer cells in CAde were higher than those in CSCC. Based on the above findings, we hypothesize that HPV infection may play greater roles in elevating stemness in CAde, resulting in a more inhibitory tumor microenvironment (e.g., less cytotoxic and more exhausted CD8^+^ T cells). HPV-positive head and neck squamous cell carcinoma could obtain more benefits from immunotherapy, whether patients with CAde can obtain such advantages compared to CSCC still needs further exploration.

Immunotherapy has emerged as a promising treatment strategy for various types of cancer, including HPV-related CC. However, the overall response rate of immunotherapy in CC is relatively low, and the efficacy differences between patients with CSCC and CAde receiving Pembrolizumab remain unknown due to limited CAde samples were included in reported clinical trials.^4^^,^^5^ It is gradually recognized that understanding the immune microenvironment of CC is the way to develop effective immunotherapeutic approaches.^73^ One approach that has shown promise in clinical trials for CC is immune checkpoint inhibitors, which block the inhibitory signals that promote cancer cells to evade the immune system.^74^ In our results, CSCC showed a higher cytotoxic score and lower exhausted score compared to CAde, which suggests the efficacy of immune checkpoint inhibitors in CSCC. Other potential immunotherapeutic approaches for CC include cancer vaccines and adoptive cell therapy.^75^ Cancer vaccines aim to stimulate the immune system to recognize and attack cancer cells with foreign antigens or neoantigens.^76^ Immunization with foreign antigens (i.e., E6 and E7) might be a good target for the host's immune system. As the study shows, the expression levels of HPV-related genes (L1, L2, E1, E2, E5, E6, and E7) were significantly higher in cancer cells both in CSCC and CAde. Adoptive cell therapy involves using a patient's immune cells that have been modified to recognize and kill cancer cells, such as tumor infiltrating leukocytes (TILs).^77^ However, as the study shows, CAde had a more inhibitory immune microenvironment, which could limit the efficacy of adoptive cell therapy in this subtype of CC.

In addition to immune cells, CAFs also play a critical role in the tumor microenvironment. CAFs are known to promote tumor growth and metastasis.^78^ This study identified that the subset of dCAFs was specifically enriched in CAde, while the subsets of vCAFs and myoCAFs were more abundant in CSCC. In addition, vCAFs and myoCAFs were negatively correlated with over survival of patients with CC, which was consistent with previous findings that the different influence of CAFs on the malignant phenotypes of tumors was responsible for the existence of subpopulations of CAFs with distinct functions.^47^ Our results confirmed that primary fibroblasts from CSCC displayed more pro-metastatic potential. Furthermore, CAFs could interact with immune cells as well as other immune components within the tumor microenvironment via secreting various cytokines, chemokines, and other effectors, which consequently shapes an immunosuppressive tumor microenvironment that also promotes cancer cells to evade the immune system.^79^ In our results, NRG1-ERBB2 and FN1-ITGA3 were identified as the specific L-R interaction pairs between CAFs and cancer cells for CSCC and CAde. Previous studies have found that NRG1-ERBB3/ERBB2 axis triggers anchorage-independent growth of basal-like breast cancer cells.^80^ The transcription levels of NRG1 act in an autocrine fashion through an ERBB2/4 heterodimer to promote the invasion of pancreatic stellate cells.^81^ Integrin, as a key transmembrane receptor, has been proven to play key roles in the development and carcinogenesis of malignant tumors by regulating MAPK or STAT5.^67^^,^^68^^,^^82^ Consistent with the results of cellular communications, some specifically sensitive chemotherapy drugs targeting ERBB2 and MAPK/STAT5 were identified for CAde and CSCC, respectively.

Although a wealth of information has been brought by this study attempting to distinguish molecular differences between the two subtypes of CC based on snRNA-seq, there are some limitations. Firstly, it remains unclear whether each cell population is preserved across cancer types. The second limitation is the lack of direct experiments underlying the phenotypes related to the different molecular expression patterns. Finally, further studies are needed to elucidate the role of these CAFs in CSCC and their potential as therapeutic targets.

## Contributors

PW, GL and DM were responsible for the study conception and design; SL, YX, BL, BH, TP, WZ, MX, WD, and FR collected clinical samples and performed laboratory experiments; SL, YS and ZZ processed data and performed bioinformatic analysis; SL, YS, CC and ZZ wrote the manuscript. SL and PW have directly accessed and verified the underlying data reported in this manuscript. All authors have read and approved the final manuscript.

## Data sharing statement

The snRNA-seq data of CAde samples has been uploaded to the Genome Sequence Archive (GSA) of the National Genomics Data Center (Access Link: https://ngdc.cncb.ac.cn/gsa-human/browse/; ID: HRA004408). The snRNA-seq data of CSCC samples has been previously deposited into the CNSA (CNGB Sequence Archive) of CNGBdb (Access link: https://db.cngb.org/cnsa/; ID: CNP0002535).

## Declaration of interests

The authors declare that they have no competing interests.

## Appendix A Supplementary data

Supplementary Table S14

SiHa-STR validation

ME-180-STR validation

HUVEC-STR validation

HUVEC-Cross Contamination-STR

HeLa-STR validation

CaSki-STR validation
