# Supplementary material for: Single-nucleus RNA sequencing reveals heterogenous microenvironments and specific drug response between cervical squamous cell carcinoma and adenocarcinoma
Source: eBioMedicine. 2023 Oct 24;97:104846. doi: 10.1016/j.ebiom.2023.104846 (PMC10618708; doi:10.1016/j.ebiom.2023.104846)

中国典型培养物保藏中心

CHINA CENTER FOR TYPE CULTURE COLLECTION (CCTCC)

Wuhan University, Wuhan 430072, China

Phone: 86-027-68752093 **Fax:** 86-027-68754833 **Email:** shenchao@whu.edu.cn

07-21-2023

Entrusted by Tongji Hospital, Tongji Medical College, Hua Zhong University of Science and Technology, CCTCC has conducted identification experiments on the SiHa, and come to the following conclusions:

1. There was no third allele found in SiHa, it indicating that there was no cross-contaminant of human source cell.
2. Compared the STR data of SiHa cell line in the databases of ATCC, DSMZ and CELLOSAURUS, all the loci of SiHa were exactly matched with the loci of SiHa (HTB-35) cells found in ATCC, DSMZ and CELLOSAURUS cell databases.

Manager：

China Center for Type Culture Collection

Note:

1. The result is only responsible for the test sample, and the genomic DNA will be reserved for three months.
2. Reference of human cell authentication: ANSI/ATCC ASN-0002-2021

Table 1. The alleles of 21 loci in SiHa

SiHa（Fig. No. SJ-01070）		
Marker	Allele 1	Allele 2
D19S433	14.2	14.2
D5S818	9	9
D21S11	29	31
D18S51	15	15
D6S1043	18	18
AMEL	X	X
D3S1358	16	17
D13S317	11	11
D7S820	10	10
D16S539	12	12
CSF1PO	12	12
Penta D	9	12
D2S441	10	10
vWA	14	17
D8S1179	13	16
TPOX	8	8
Penta E	10	12
TH01	6	9
D12S391	19	22
D2S1338	24	24
FGA	21	21

CCTCC

Sample Name	Panel	SQ
sj01070	MR21_v2.1	

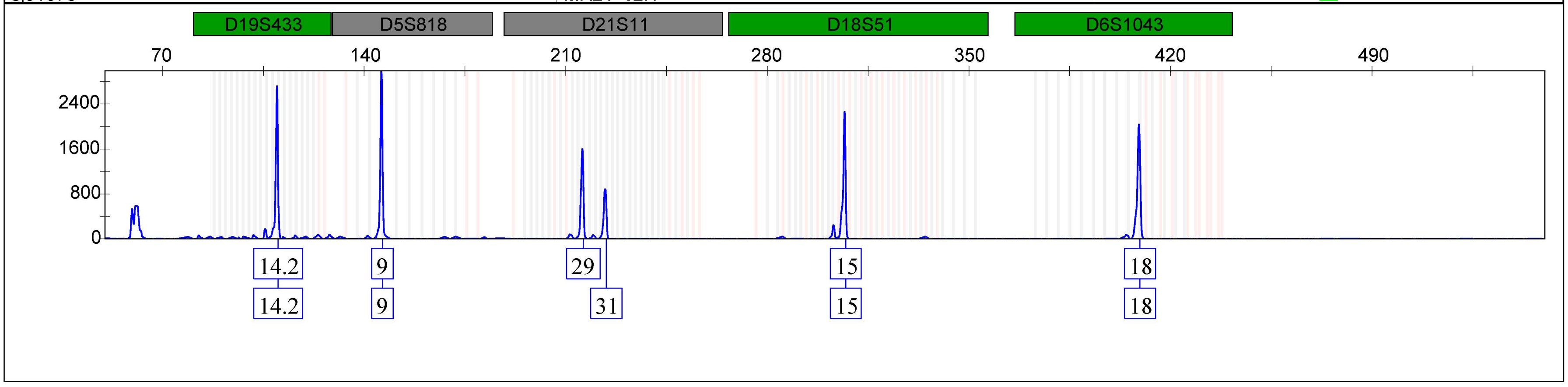

sj01070	MR21_v2.1	

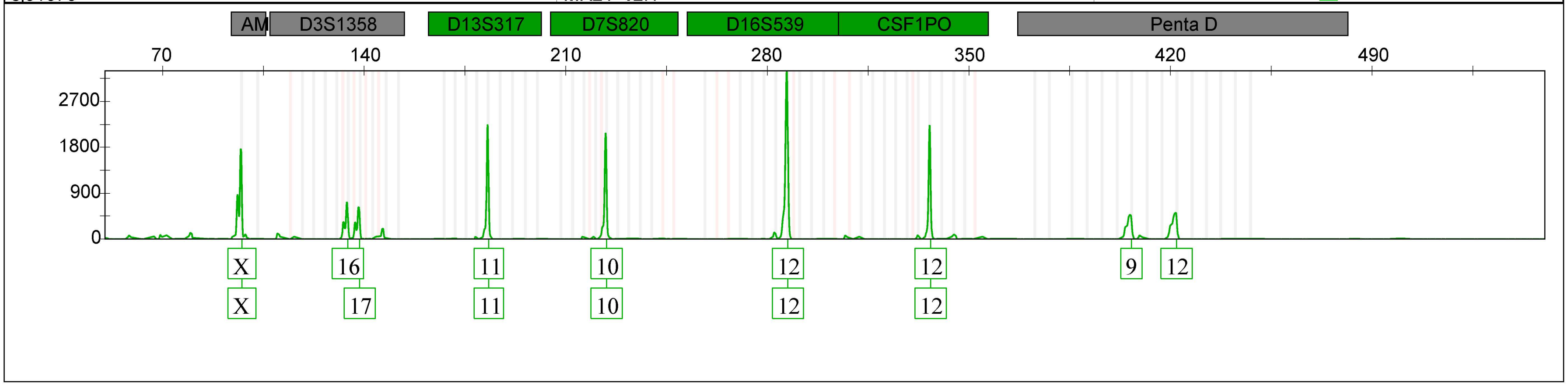

sj01070	MR21_v2.1	

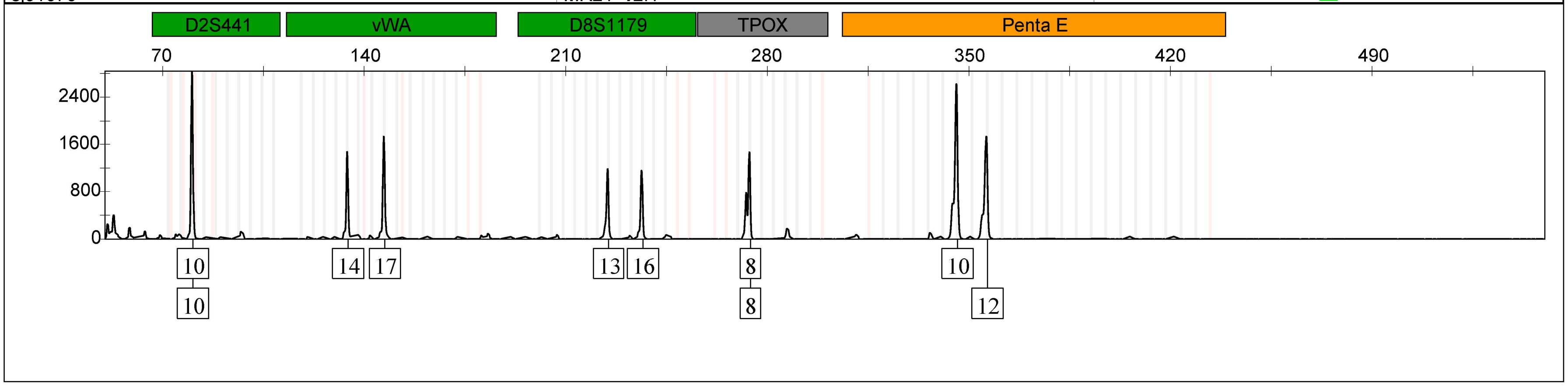

sj01070	MR21_v2.1	

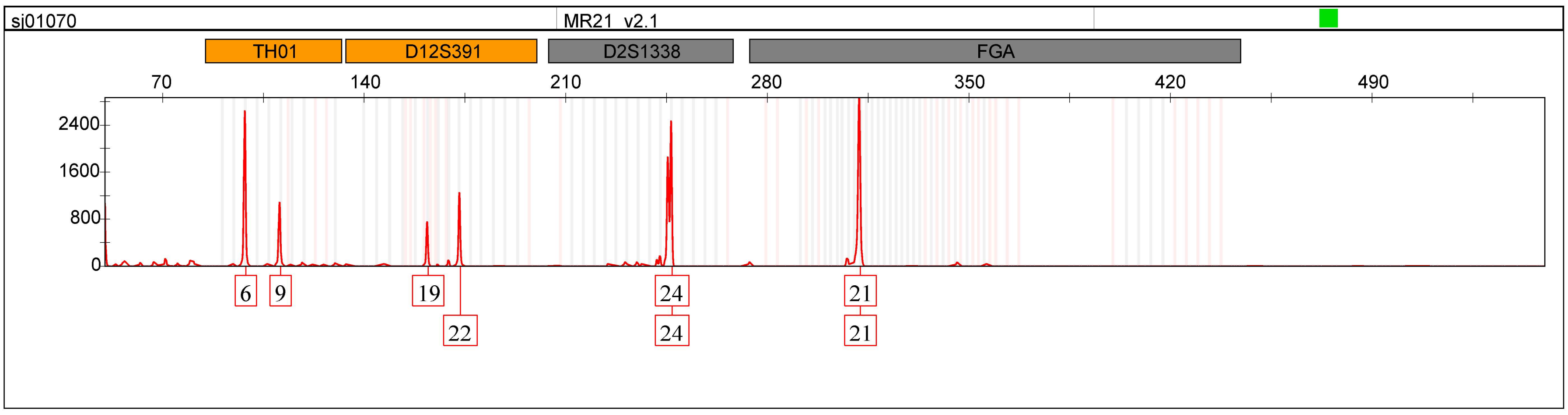

Supplement: SiHa-STR validation [file mmc15.pdf]
