# Supplementary material for: Single-nucleus RNA sequencing reveals heterogenous microenvironments and specific drug response between cervical squamous cell carcinoma and adenocarcinoma
Source: eBioMedicine. 2023 Oct 24;97:104846. doi: 10.1016/j.ebiom.2023.104846 (PMC10618708; doi:10.1016/j.ebiom.2023.104846)

中国典型培养物保藏中心

CHINA CENTER FOR TYPE CULTURE COLLECTION (CCTCC)

Wuhan University, Wuhan 430072, China

Phone: 86-027-68752093 **Fax:** 86-027-68754833 **Email:** shenchao@whu.edu.cn

07-21-2023

Entrusted by Tongji Hospital, Tongji Medical College, Hua Zhong University of Science and Technology, CCTCC has conducted identification experiments on the ME-180, and come to the following conclusions:

1. There was no third allele found in ME-180, it indicating that there was no cross-contaminant of human source cell.
2. Compared the STR data of ME-180 cell line in the databases of ATCC, DSMZ and CELLOSAURUS, all the loci of ME-180 were exactly matched with the loci of ME-180 (HTB-33) cells found in ATCC, DSMZ and CELLOSAURUS cell databases.

Manager：

中国典型培养物保藏中心 业务专用章

China Center for Type Culture Collection

Note:

1. The result is only responsible for the test sample, and the genomic DNA will be reserved for three months.
2. Reference of human cell authentication: ANSI/ATCC ASN-0002-2021

Table 1. The alleles of 21 loci in ME-180

ME-180（Fig. No. SJ-01073）		
Marker	Allele 1	Allele 2
D19S433	13	15.2
D5S818	12	12
D21S11	30	31
D18S51	12	12
D6S1043	11	11
AMEL	X	X
D3S1358	16	16
D13S317	11	13
D7S820	9	10
D16S539	12	13
CSF1PO	11	11
Penta D	9	14
D2S441	11	11
vWA	15	17
D8S1179	14	14
TPOX	8	10
Penta E	12	14
TH01	8	9.3
D12S391	18	20
D2S1338	18	18
FGA	23	23

CCTCC

GeneMapper® ID-X 1.5

Project: 20230511

Sample Name	Panel	SQ
sj01073	MR21 v2.1	

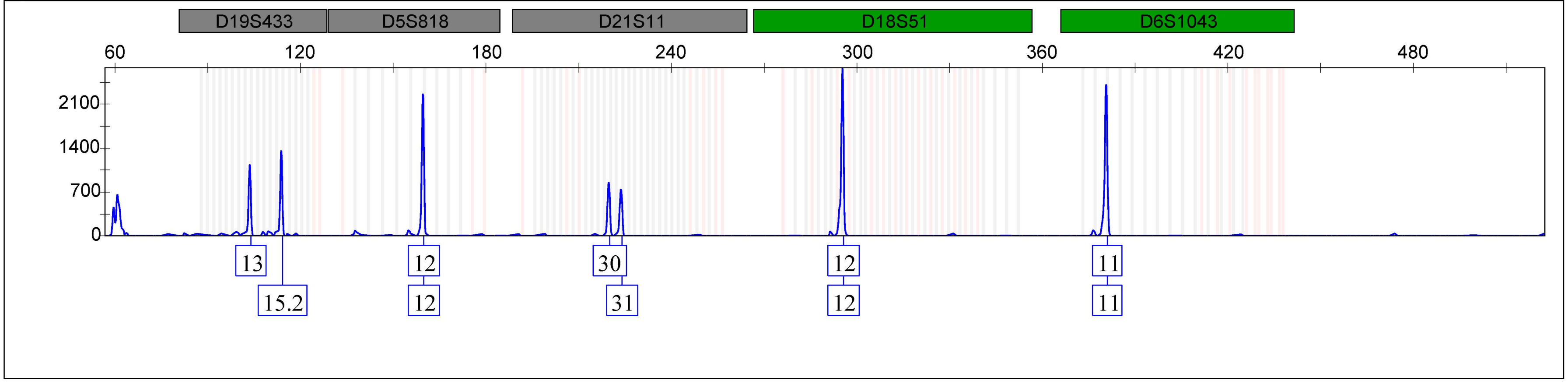

sj01073	MR21 v2.1	

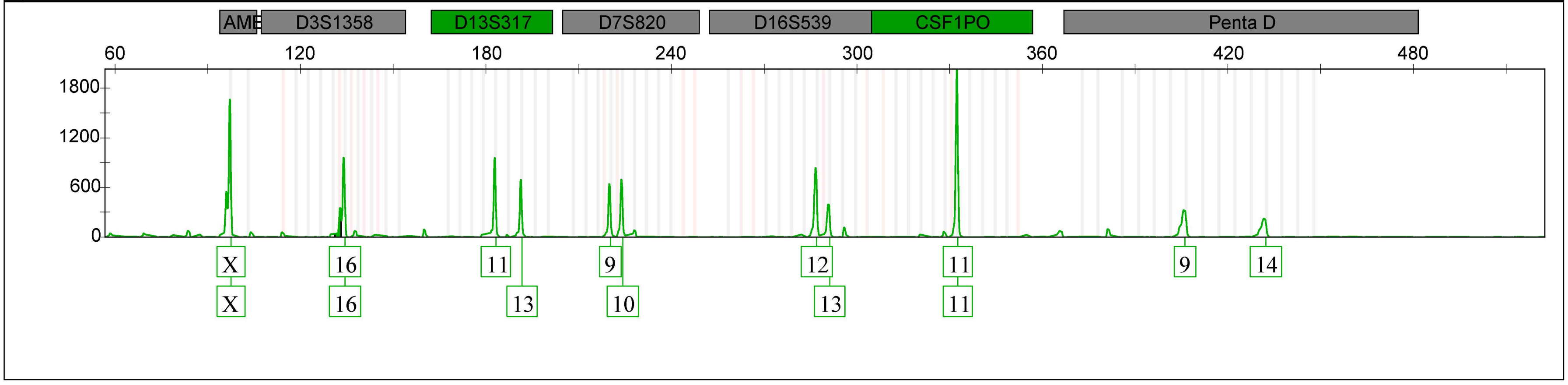

sj01073	MR21 v2.1	

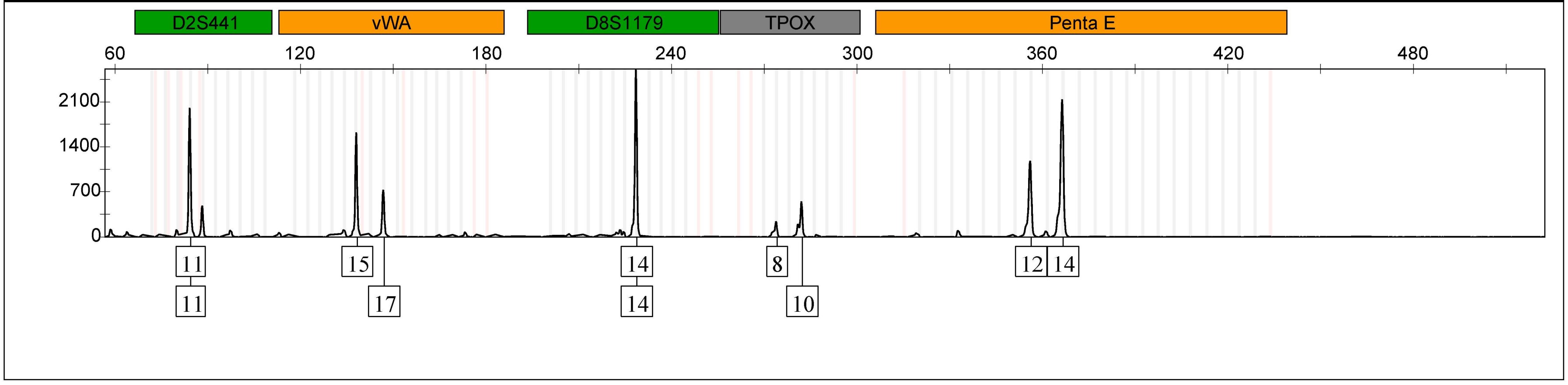

sj01073	MR21 v2.1	

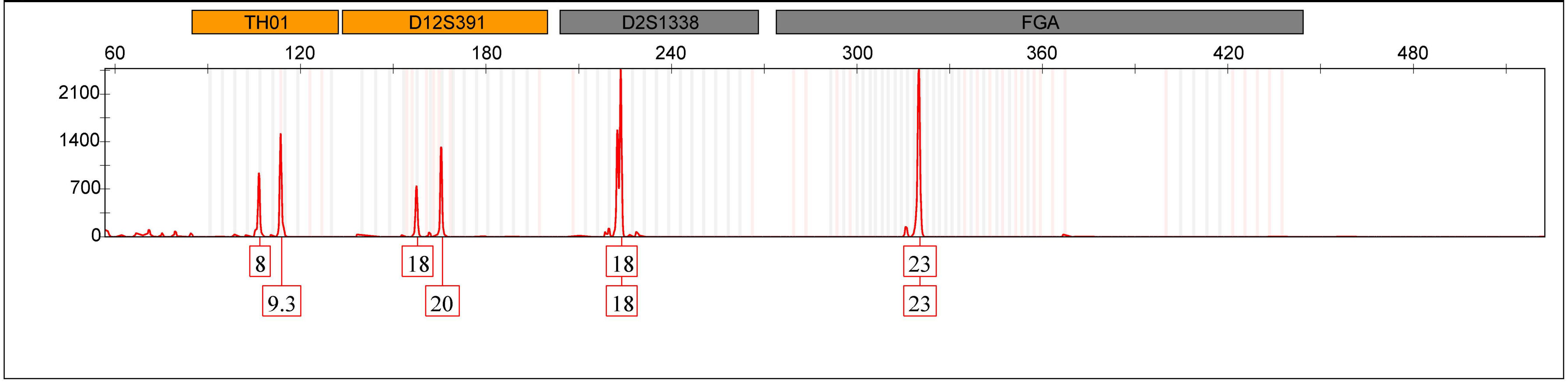

Supplement: ME-180-STR validation [file mmc16.pdf]
