# Supplementary material for: Single-nucleus RNA sequencing reveals heterogenous microenvironments and specific drug response between cervical squamous cell carcinoma and adenocarcinoma
Source: eBioMedicine. 2023 Oct 24;97:104846. doi: 10.1016/j.ebiom.2023.104846 (PMC10618708; doi:10.1016/j.ebiom.2023.104846)
Supplement: HUVEC-STR validation [file mmc17.pdf]

Cell Line Authentication Service

STR Profiling Report

Sample From: Aoyinbio, SAc0177, HUVEC

Sample Type: Cell Line

Testing Method: STR Genotyping

Report Time: August 23, 2022

Add: Room 502, NO.1015 Longteng Rd , Songjiang District, Shanghai/ Tel:+86-18521538068
Homepage: http://www.biowing.com.cn/ E-mail: market@biowing.com.cn

COMPANY STATEMENT

1. THIS REPORT IS ONLY RESPONSIBLE FOR THE SAMPLES ANALYZED.
2. THE TESTING RESULTS AND THE ORGANIZATION NAME WILL NOT BE USED FOR ADVERTISEMENT, COMMERCIAL EXHIBITIONS, COMMERCIAL PERFORMANCE AND OTHER COMMERCIAL ACTIVITIES.
3. OBJECTIONS SHOULD BE RAISED WITHIN FIFTEEN DAYS AFTER THE RECEIPT OF THIS REPORT.
4. THE PAPER REPORT WITH CONTENT ALTERING, ADDING OR WITHOUT THE STAMPED SEAL OF THE COMPANY ARE INVALID.

Testing Company: Shanghai Biowing Applied Biotechnology Co. Ltd

Address: Room 502, NO.1015 Longteng Rd , Songjiang District, Shanghai

Tel: +86-18521538068

Contact: Shuangning Zhu

E-mail: zhusn@biowing.com.cn

Cell Line Authentication – STR Profiling Report

Sample code

Table 1. Sample Code

Customer's code	Company Code
HUVEC, SAc0177	20220819-06

Sample Number :1

Sample Type: Cell line

Testing Type: STR

Testing Method:

DNA was extracted by a commercial kit from CORNING (AP-EMN-BL-GDNA-250G). The twenty STRs including Amelogenin locus were amplified by six multiplex PCR and separated on ABI 3730XL Genetic Analyzer. The signals were then analyzed by the software GeneMapper.

Data Interpretation:

Cell lines were authenticated using Short Tandem Repeat (STR) analysis asdescribed in 2012 in ANSI Standard (ASN-0002) by the ATCC Standards Development Organization (SDO) and in Capes-Davis et al., Match criteria for human cell line authentication: Where do we draw the line? Int J Cancer.2013;132(11):2510-9.

Add: Room 502, NO.1015 Longteng Rd , Songjiang District, Shanghai/ Tel:+86-18521538068
Homepage: http://www.biowing.com.cn/ E-mail: market@biowing.com.cn

Test Results

1. STR profile

Table 2. STR and Amelogenin Genotyping Results of Cell line.

Loci	Sample information			Control information		
	Sample name: HUVEC, SAc0177			Cell line name:		
	**Allele1**	**Allele2**	**Allele3**	**Allele1**	**Allele2**	**Allele3**
D5S818	7	8				
D13S317	9	11				
D7S820	11	11				
D16S539	9	11				
VWA	14	14				
TH01	7	9				
AMEL	X	X				
TPOX	8	8				
CSF1PO	12	14				
D12S391	19	20				
FGA	21	25				
D2S1338	22	24				
D21S11	30	31				
D18S51	13	19				
D8S1179	12	15				
D3S1358	15	18				
D6S1043	18	19				
PENTAE	11	16				
D19S433	15.2	15.2				
PENTAD	11	12				
D1S1656	15	17				

Add: Room 502, NO.1015 Longteng Rd , Songjiang District, Shanghai/ Tel:+86-18521538068
Homepage: http://www.biowing.com.cn/ E-mail: market@biowing.com.cn

2. database annotation

Figure 1. STR matching analysis

EV	Cell No.	Cell name	Locus names								
			D5S818	D13S317	D7S820	D16S539	VWA	TH01	AM	TPOX	CSF1PO
	Query (Your Cell)		7,8	9,11	11,11	9,11	14,14	7,9	X,X	8,8	12,14

Note: The STR online match analysis of the test cell against DSMZ database, showing cell number (Cell No.) and cell name.

3. Authentication

☑ The submitted sample profile is human, but not a match for any profile in the DSMZ. As the STR database of Immortalized primary cells **HUVEC** was not logged in, the matching result can't be displayed. The cell line is unique and not cross-contaminated or misidentified, if the paper was to publish, these data could be submitted to the magazine.

☐ The submitted profile is an exact match for the following human cell line(s) in the DSMZ STR database (8 core loci plus Amelogenin): .

☐ The submitted profile is similar to the following DSMZ human cell line: /.

- **Note:** A cell line can be considered to be authenticated when 80% (exact match) of the alleles in its STR profile match profiles from tissue or other cell line samples from that donor or from database. Cell lines with between a 55% to 80% (similar) match require further profiling for investigation of relatedness.

Figure 2. STR profiles of sample cell line

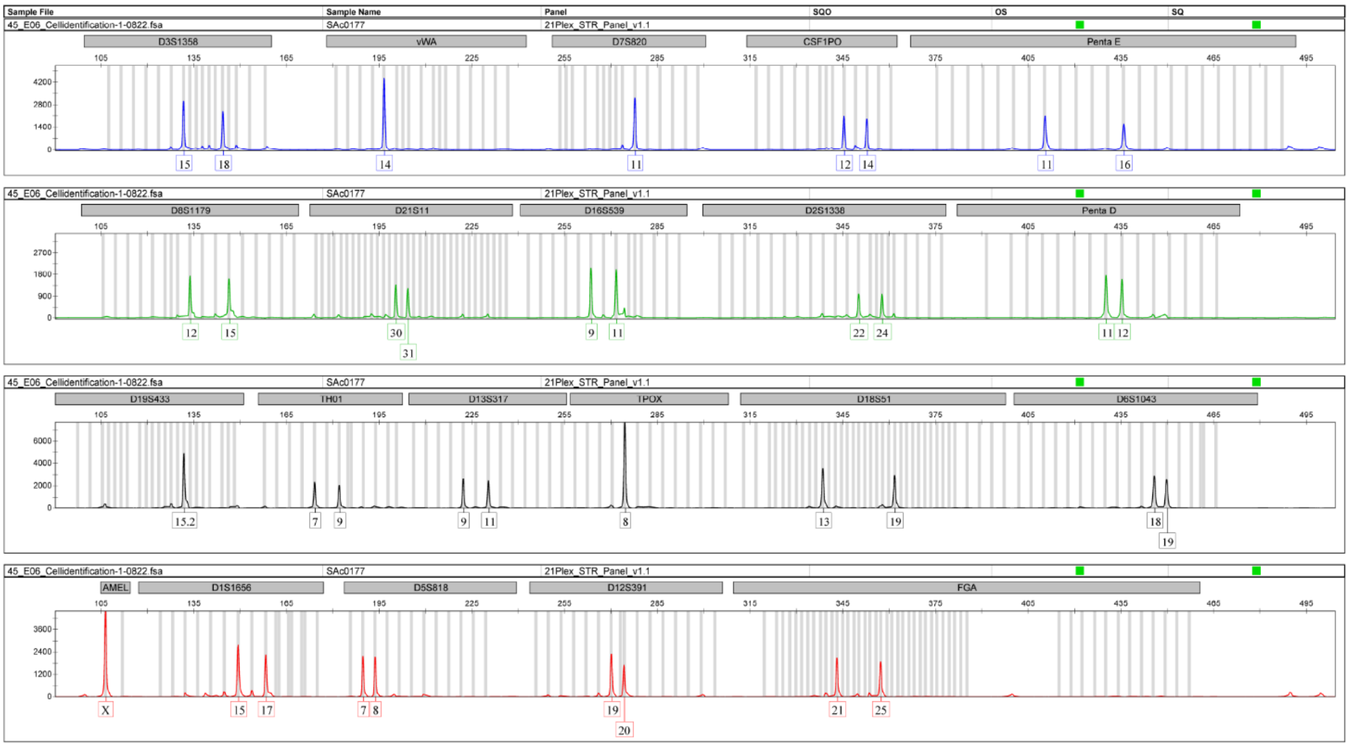

Add: Room 502, NO.1015 Longteng Rd , Songjiang District, Shanghai/ Tel:+86-18521538068
Homepage: http://www.biowing.com.cn/ E-mail: market@biowing.com.cn

Appendix

1. Genotyping Strategy and Site Distribution

Table S1. Experimental Strategy and Sites

	Strategy 1	Strategy 2	Strategy 3	Strategy 4
1	D3S1358	D8S1179	D19S433	AMEL
2	VWA	D21S11	TH01	D1S1656
3	D7S820	D16S539	D13S317	D5S818
4	CSF1PO	D2S1338	TPOX	D12S391
5	PENTAE	PENTAD	D18S51	FGA
6			D6S1043	

The allele match algorithm compares the 8 core loci plus amelogenin only, even though alleles from all lociwill be reported when available.

2. DSMZ tools was used to carry on the cell line comparison, which contains 2455 cell lines STR data from ATCC, DSMZ, JCRB ,ECACC， GNE and RIKEN databases. If the cell is not included in the above cell library, users need to compared with other databases.

Shanghai Biowing Applied Biotechnology Co. Ltd * STR Profiling Cell Line Authentication

Technician: Jianan Zhang

Checked by: Ning Qian

Issued by: Yang Bai

Issue date: August 23, 2022

Add: Room 502, NO.1015 Longteng Rd , Songjiang District, Shanghai/ Tel:+86-18521538068
Homepage: http://www.biowing.com.cn/ E-mail: market@biowing.com.cn
