# Supplementary material for: Single-nucleus RNA sequencing reveals heterogenous microenvironments and specific drug response between cervical squamous cell carcinoma and adenocarcinoma
Source: eBioMedicine. 2023 Oct 24;97:104846. doi: 10.1016/j.ebiom.2023.104846 (PMC10618708; doi:10.1016/j.ebiom.2023.104846)

中国典型培养物保藏中心

CHINA CENTER FOR TYPE CULTURE COLLECTION (CCTCC)

Wuhan University, Wuhan 430072, China

Phone: 86-027-68752093 **Fax:** 86-027-68754833 **Email:** shenchao@whu.edu.cn

08-17-2023

Entrusted by Tongji Hospital, Tongji Medical College, Hua Zhong University of Science and Technology, CCTCC has conducted identification experiments on the HUVEC, and come to the following conclusions:

1. There were four third alleles found in D21S11，D18S51 locus of HUVEC cell line, it indicating that there was no cross-contaminant of human source cell line.
2. Compared the STR data of HUVEC in the databases of ATCC, DSMZ and CELLOSAURUS, its profile does not exactly match with any of the current data (Table 1).
3. The STR data of HUVEC and SK-OV-3 (HTB-77) cell matches the highest rate of 88.14% in CELLOSAURUS database.

Manager:

China Center for Type Culture Collection

Note:

1. The result is only responsible for the test sample, and the genomic DNA will be reserved for three months.
2. Reference of human cell authentication: ANSI/ATCC ASN-0002-2021

Table 1. The alleles of 21 loci in HUVEC

HUVEC（Fig. No. SJ-01081）			
Marker	Allele 1	Allele 2	Allele 3
D19S433	14	14.2	
D5S818	11	12	
D21S11	30	31	31.2
D18S51	16	17	18
D6S1043	12	12	
AMEL	X	X	
D3S1358	14	14	
D13S317	8	11	
D7S820	14	14	
D16S539	12	12	
CSF1PO	11	11	
Penta D	13	13	
D2S441	10	11.3	
vWA	18	18	
D8S1179	14	14	
TPOX	11	11	
Penta E	5	13	
TH01	9	9.3	
D12S391	22	22	
D2S1338	17	23	
FGA	24	25	

CCTCC

Applied Biosystems
GeneMapper® ID-X 1.5

Project: 20230511

Sample Name	Panel	SQ
SJ-01081	MR21 v2.1	

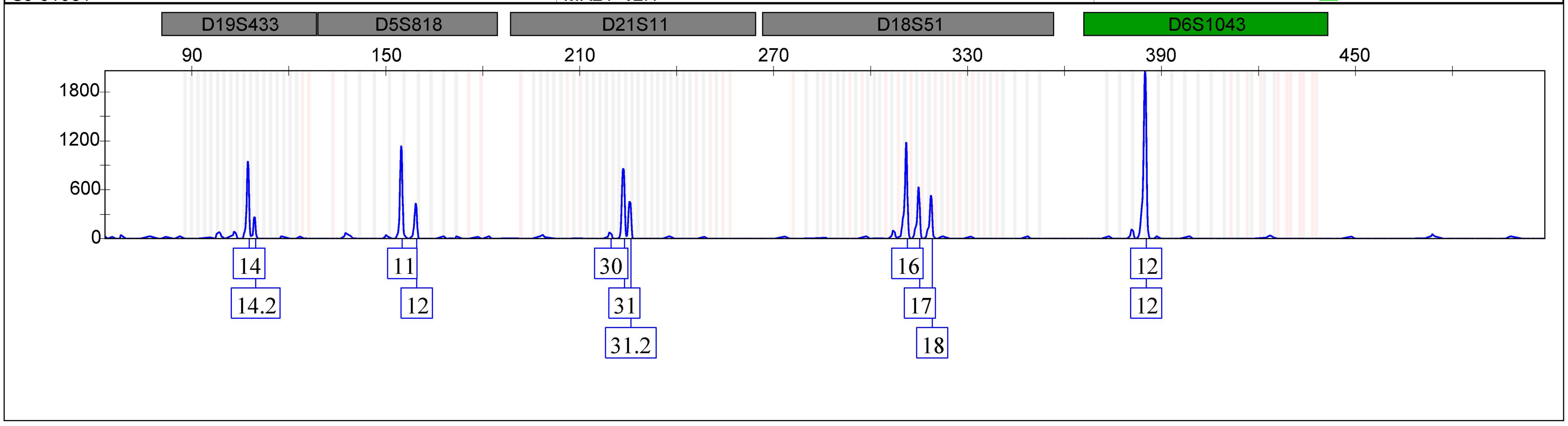

SJ-01081	MR21 v2.1	

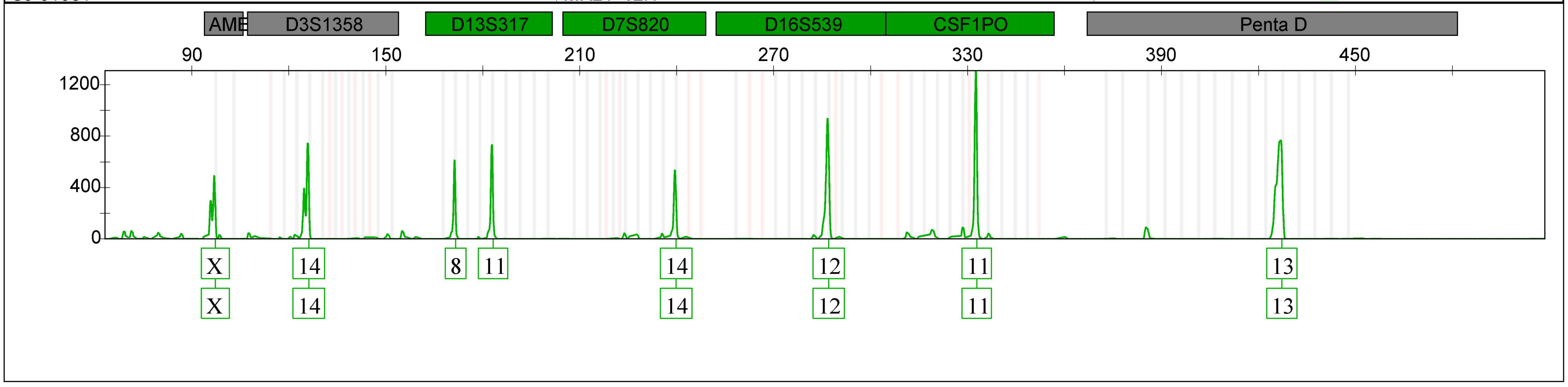

SJ-01081	MR21 v2.1	

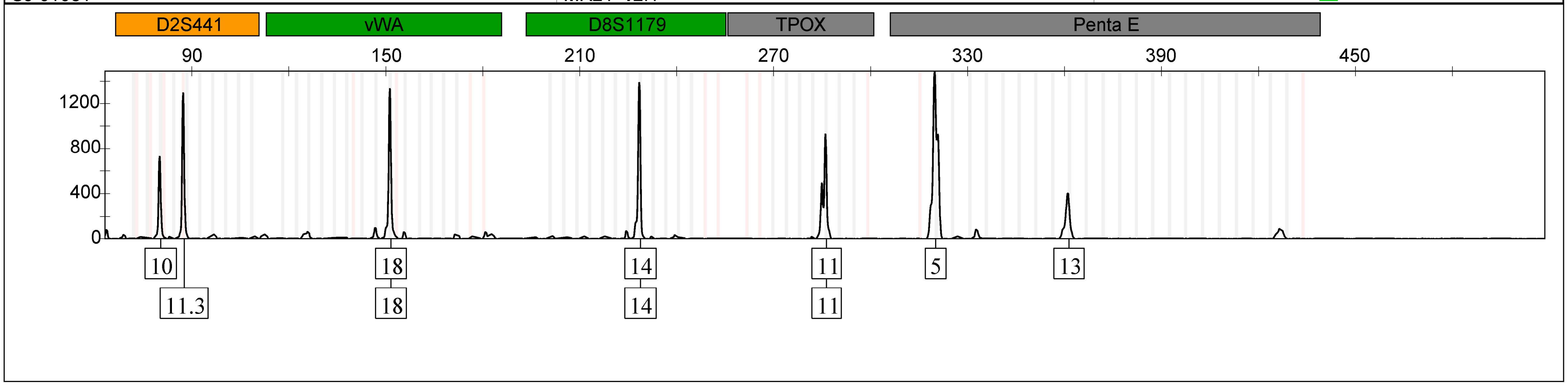

SJ-01081	MR21 v2.1	

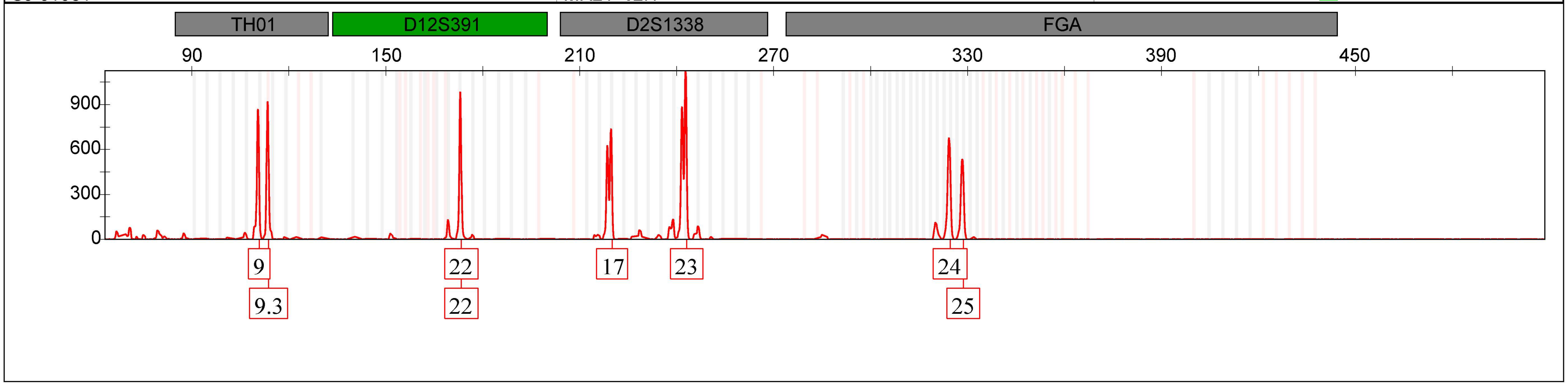

Supplement: HUVEC-Cross Contamination-STR [file mmc18.pdf]
