# Supplementary material for: Single-nucleus RNA sequencing reveals heterogenous microenvironments and specific drug response between cervical squamous cell carcinoma and adenocarcinoma
Source: eBioMedicine. 2023 Oct 24;97:104846. doi: 10.1016/j.ebiom.2023.104846 (PMC10618708; doi:10.1016/j.ebiom.2023.104846)

中国典型培养物保藏中心

CHINA CENTER FOR TYPE CULTURE COLLECTION (CCTCC)

Wuhan University, Wuhan 430072, China

Phone: 86-027-68752093 **Fax:** 86-027-68754833 **Email:** shenchao@whu.edu.cn

07-21-2023

Entrusted by Tongji Hospital, Tongji Medical College, Hua Zhong University of Science and Technology, CCTCC has conducted identification experiments on the HeLa, and come to the following conclusions:

1. There was no third allele found in HeLa, it indicating that there was no cross-contaminant of human source cell.
2. Compared the STR data of HeLa cell line in the databases of ATCC, DSMZ and CELLOSAURUS, all the loci of HeLa were exactly matched with the loci of HeLa (CCL-2) cells found in ATCC, DSMZ and CELLOSAURUS cell databases.

Manager：

China Center for Type Culture Collection

Note:

1. The result is only responsible for the test sample, and the genomic DNA will be reserved for three months.
2. Reference of human cell authentication: ANSI/ATCC ASN-0002-2021

Table 1. The alleles of 21 loci in HeLa

HeLa（Fig. No. SJ-01071）		
Marker	Allele 1	Allele 2
D19S433	13	14
D5S818	11	12
D21S11	27	28
D18S51	16	16
D6S1043	18	18
AMEL	X	X
D3S1358	15	18
D13S317	12	13.3
D7S820	8	12
D16S539	9	10
CSF1PO	9	10
Penta D	8	15
D2S441	10	11
vWA	16	18
D8S1179	12	13
TPOX	8	12
Penta E	7	17
TH01	7	7
D12S391	20	25
D2S1338	17	17
FGA	18	21

CCTCC

Sample Name	Panel	SQ
sj01071	MR21_v2.1	

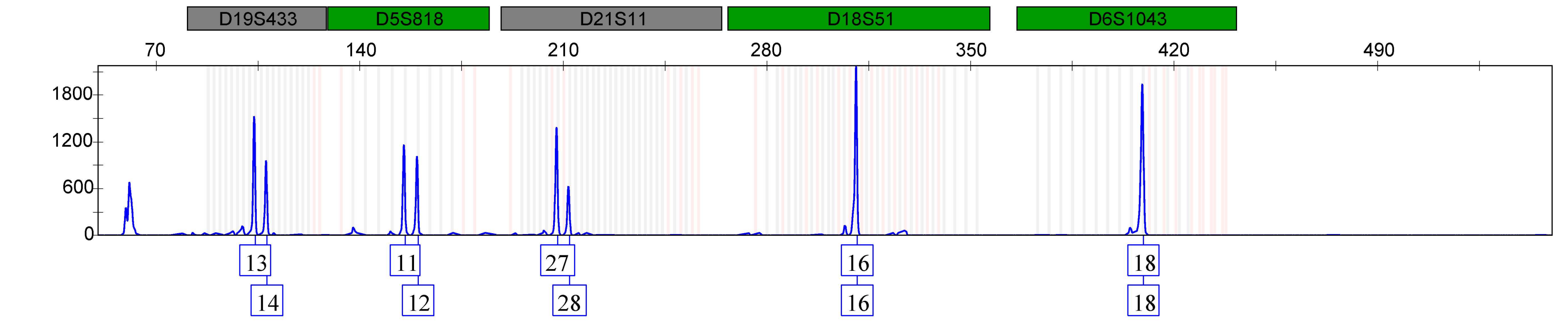

sj01071	MR21_v2.1	

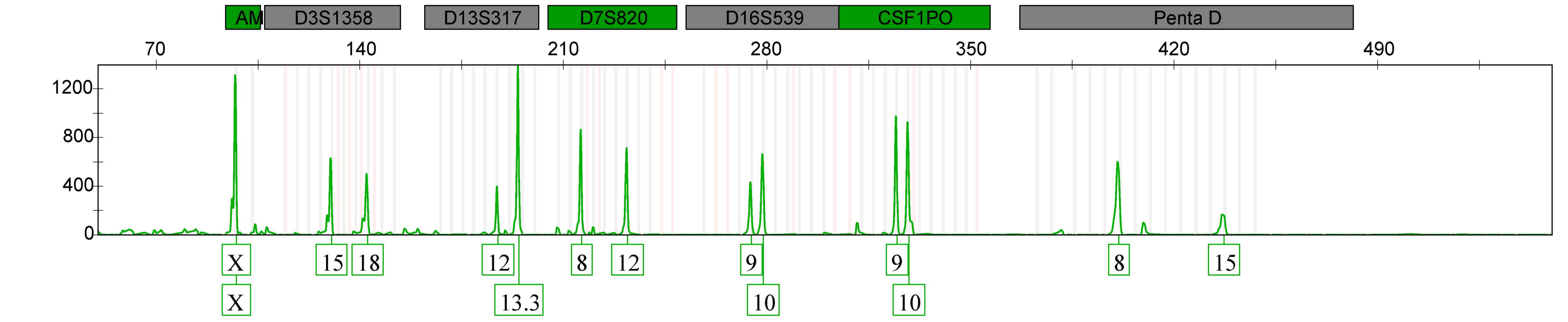

sj01071	MR21_v2.1	

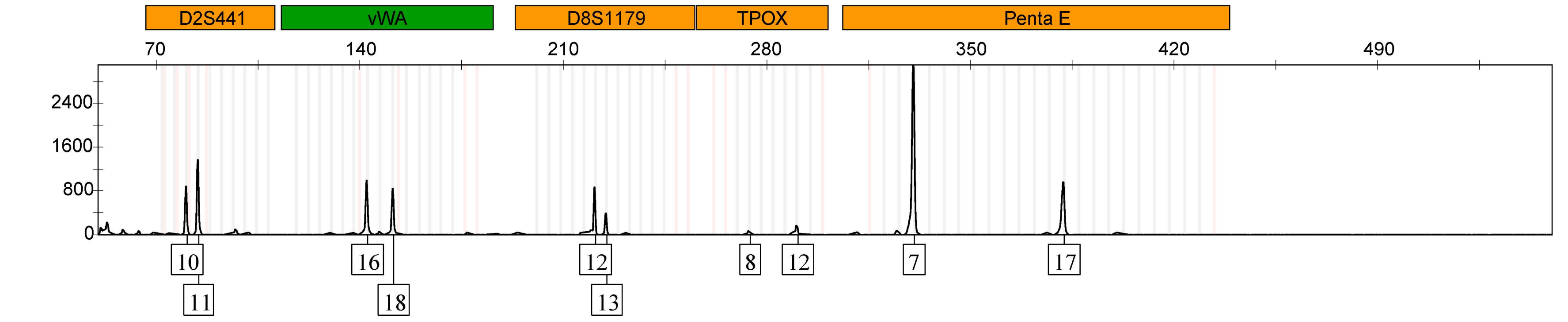

sj01071	MR21_v2.1	

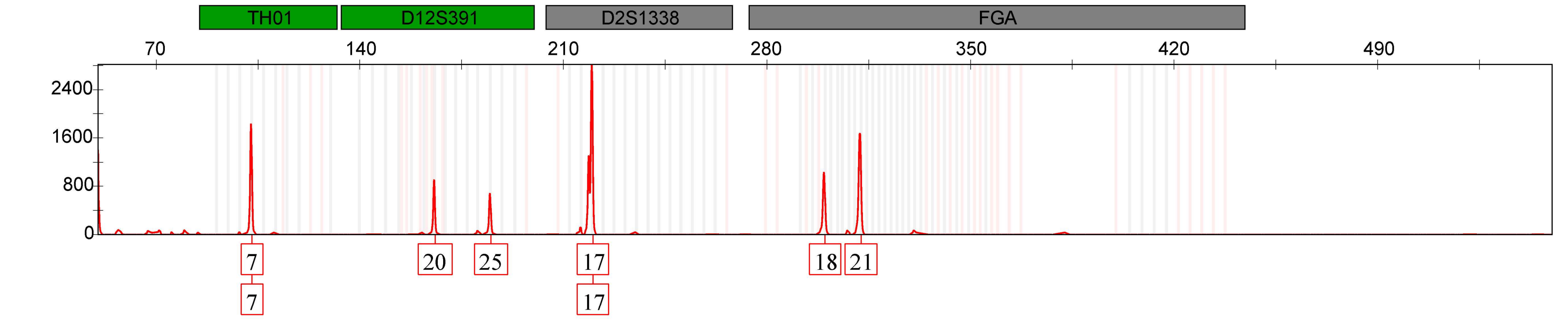

Supplement: HeLa-STR validation [file mmc19.pdf]
