# Supplementary material for: Single-nucleus RNA sequencing reveals heterogenous microenvironments and specific drug response between cervical squamous cell carcinoma and adenocarcinoma
Source: eBioMedicine. 2023 Oct 24;97:104846. doi: 10.1016/j.ebiom.2023.104846 (PMC10618708; doi:10.1016/j.ebiom.2023.104846)

中国典型培养物保藏中心

CHINA CENTER FOR TYPE CULTURE COLLECTION (CCTCC)

Wuhan University, Wuhan 430072, China

Phone: 86-027-68752093 **Fax:** 86-027-68754833 **Email:** shenchao@whu.edu.cn

07-21-2023

Entrusted by Tongji Hospital, Tongji Medical College, Hua Zhong University of Science and Technology, CCTCC has conducted identification experiments on the CaSki, and come to the following conclusions:

1. There was no third allele found in CaSki, it indicating that there was no cross-contaminant of human source cell.
2. Compared the STR data of CaSki cell line in the databases of ATCC, DSMZ and CELLOSAURUS, all the loci of CaSki were exactly matched with the loci of CaSki (CRL-1550) cells found in ATCC, DSMZ and CELLOSAURUS cell databases.

Manager：

China Center for Type Culture Collection

中国典型培养物保藏中心 业务专用章

Note:

1. The result is only responsible for the test sample, and the genomic DNA will be reserved for three months.
2. Reference of human cell authentication: ANSI/ATCC ASN-0002-2021

Table 1. The alleles of 21 loci in CaSki

CaSki（Fig. No. SJ-01072）		
Marker	Allele 1	Allele 2
D19S433	15	16
D5S818	13	13
D21S11	30	30
D18S51	17	17
D6S1043	11	14
AMEL	X	X
D3S1358	15	15
D13S317	8	12
D7S820	8	11
D16S539	11	12
CSF1PO	10	10
Penta D	10	10
D2S441	11	11
vWA	17	17
D8S1179	15	15
TPOX	8	8
Penta E	5	5
TH01	7	7
D12S391	18	18
D2S1338	21	21
FGA	21	21

CCTCC

Sample Name	Panel	SQ
sj01072	MR21_v2.1	

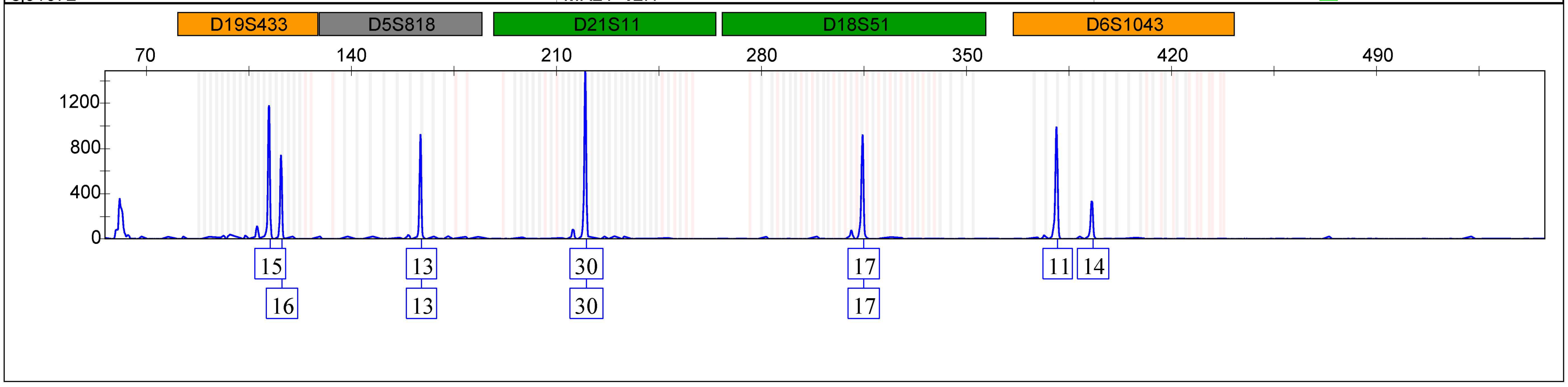

sj01072	MR21_v2.1	

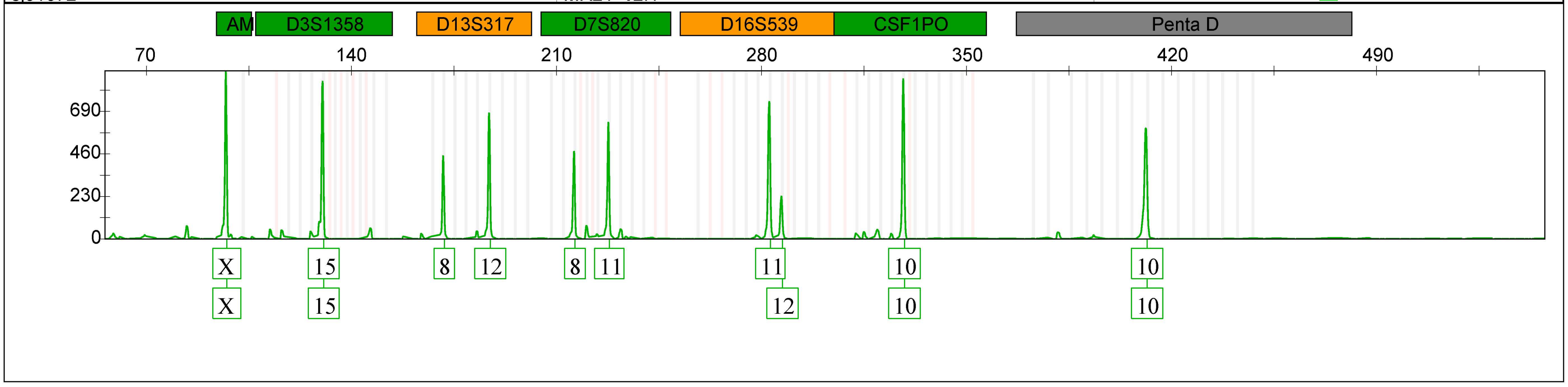

sj01072	MR21_v2.1	

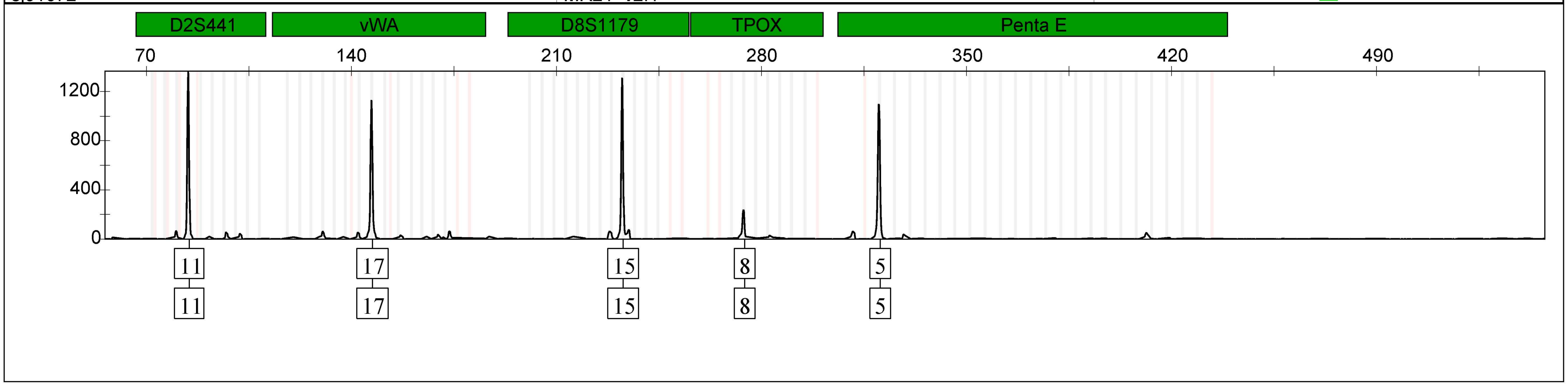

sj01072	MR21_v2.1	

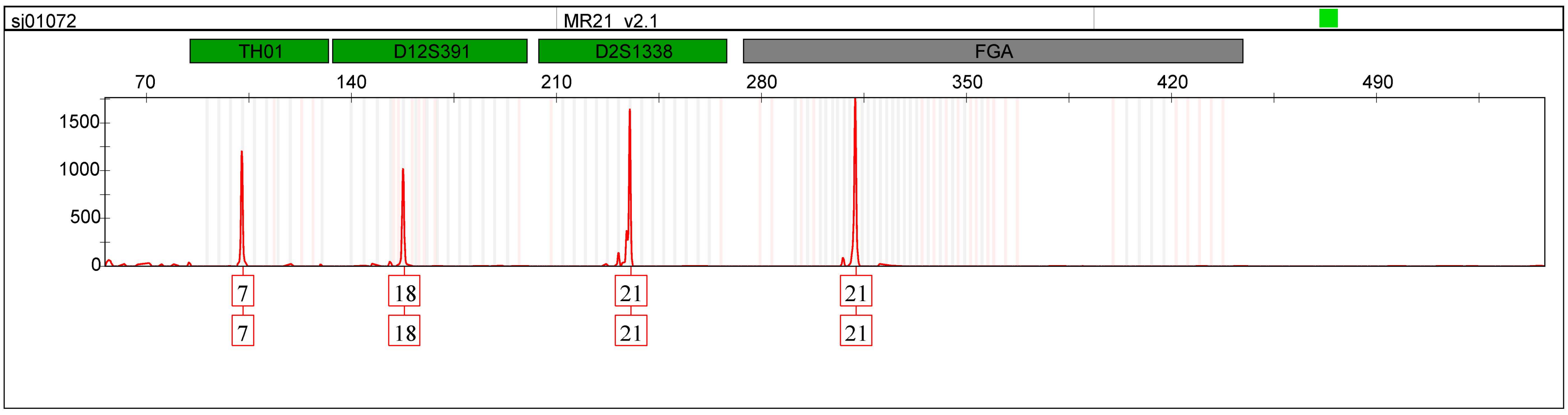

Supplement: CaSki-STR validation [file mmc20.pdf]
